# Vibrissa Self-Motion and Touch Are Reliably Encoded along the Same Somatosensory Pathway from Brainstem through Thalamus

**DOI:** 10.1371/journal.pbio.1002253

**Published:** 2015-09-22

**Authors:** Jeffrey D. Moore, Nicole Mercer Lindsay, Martin Deschênes, David Kleinfeld

**Affiliations:** 1 Department of Physics, University of California, San Diego, La Jolla, California, United States of America; 2 Section of Neurobiology, University of California, San Diego, La Jolla, California, United States of America; 3 Centre de Recherche Université Laval Robert-Giffard, Québec City, Québec, Canada; ICM - Institut du Cerveau et de la Moelle épinière - Hôpital Pitié-Salpêtrière 47, FRANCE

## Abstract

Active sensing involves the fusion of internally generated motor events with external sensation. For rodents, active somatosensation includes scanning the immediate environment with the mystacial vibrissae. In doing so, the vibrissae may touch an object at any angle in the whisk cycle. The representation of touch and vibrissa self-motion may in principle be encoded along separate pathways, or share a single pathway, from the periphery to cortex. Past studies established that the spike rates in neurons along the lemniscal pathway from receptors to cortex, which includes the principal trigeminal and ventral-posterior-medial thalamic nuclei, are substantially modulated by touch. In contrast, spike rates along the paralemniscal pathway, which includes the rostral spinal trigeminal interpolaris, posteromedial thalamic, and ventral zona incerta nuclei, are only weakly modulated by touch. Here we find that neurons along the lemniscal pathway robustly encode rhythmic whisking on a cycle-by-cycle basis, while encoding along the paralemniscal pathway is relatively poor. Thus, the representations of both touch and self-motion share one pathway. In fact, some individual neurons carry both signals, so that upstream neurons with a supralinear gain function could, in principle, demodulate these signals to recover the known decoding of touch as a function of vibrissa position in the whisk cycle.

## Abstract 2

Recordings from behaving rats show that information regarding the position of whiskers during "whisking" is encoded along the same neuronal pathway as touch. This contrasts with the separate pathways used for encoding position and touch in the limbs and implies the need for non-linear demodulation of touch with respect to whisker position.

## Author Summary

Animals interrogate the world around them with actively moving sensory organs, resulting in a blend of sensory inputs: one input is from the object under study, while the second is from self-generated movement of the sensor. The detection of an object thus depends on the ability of the animal to distinguish among internally versus externally generated sensations. Nervous systems employ various signaling mechanisms to reference inputs from a sensory organ relative to its position. A well-known example is proprioception, in which receptors in the limb muscles and joints are used to infer the position of tactile sensors in the hands. In this case, the signals of limb position are encoded in areas of the brain that are distinct from those encoding touch. Here, we investigate the analogous problem of encoding the position of the vibrissae, or whiskers—essential orofacial sensorimotor organs in rodents. In contrast to the case for limbs, we find that vibrissa position is encoded along the same neuroanatomical pathway as vibrissa touch. The seeming ambiguity that results from the mixed representation of position and touch can be resolved by a nonlinear neuronal input-output relation that demodulates touch with respect to vibrissa position. This scheme enables the rodent to determine where an object is located relative to its body axis.

## Introduction

Animals navigate the world around them with actively moving sensory organs [1]. This process results in a blend of sensory input from the presence of two underlying sensory signals. One input is from the environment or object under study, while the second is from self-generated movement of the sensor [2]. The detection of an external stimulus with confidence, as well as the ability to confirm the position and trajectory of the sensor, depends on the ability of the animal to distinguish among internally versus externally generated sensations. Ambiguity among these sources leads to unpleasant outcomes, such as vertigo [3] and motion sickness [4] for the case of vestibular control.

To resolve this ambiguity, nervous systems use three complementary signaling mechanisms to reference input from a sensory organ relative to the position of the sensors [5]. One is to encode self-generated sensor movement by the exo-receptors that also encode changes in the external environment; this is denoted peripheral re-afference. A second mechanism is to use muscular endo-receptors to encode elongation and contraction force, as performed by spindle fibers and Golgi tendons, respectively; this is denoted proprioception. A third mechanism is to generate a central copy of the motor commands for the intended sensor position; this is denoted corollary discharge. These three mechanisms report complementary, but not necessarily complete [6], information on sensor position.

While movement of a limb involves proprioceptive and corollary discharge reference signals, current evidence suggests that facial muscles, which bridge attachment points across soft tissue as opposed to bone, contain neither spindle fibers nor Golgi tendons [7–11]. Additional evidence demonstrates that despite the presumed lack of proprioceptors in the vibrissa musculature, neuronal signals related to rhythmic self-generated vibrissa motion, i.e. whisking, are encoded predominantly through peripheral sensory mechanisms [12–14]. Together, these observations lead to the hypothesis that self-generated vibrissa motion is encoded through re-afferent activation of mechanoreceptors. Specifically, activation of lanceolate- and/or Merkel-ending trigeminal neurons could presumably encode both re-afferent and ex-afferent input. These primary sensory neurons have identical, broad axonal arborizations across nuclei in the trigeminal brainstem [15,16]. Vibrissa self-motion signals are thought to inform the rodent about the position of its vibrissae upon tactile contact with an object [17–20], though an alternative possibility based on contact forces has been proposed [21] and critiqued [22].

How might the animal determine the location of objects that it contacts with its moving vibrissae? Past work shows that the strength of vibrissal ex-afferent touch responses, as measured in cortex, are strongly modulated by the phase in the whisk cycle at the moment of contact [20]. The responses of these units, therefore, contain the information necessary to determine object location through self-motion, but the underlying neuronal architecture required to achieve this cortical representation of object location remains unknown. Elements of signal detection theory [23] suggest two scenarios to demodulate touch relative to phase in the whisk cycle. One scenario is that the whisking and touch signals are encoded by different populations of peripheral receptors and are maintained as separate whisking and touch pathways to somatosensory cortex. A plausible scheme for demodulation involves gating of the touch signal by the separate whisking signal [20]. A second scenario is that both whisking and touch signals are encoded by the same sensory receptors and central neurons to cortex. In this case, a gain function with an accelerating nonlinearity [24] can serve to demodulate the touch signal.

As a means to gain insight into the particular scenario used by rodents to merge touch and self-motion of the vibrissae, we examine the response of neurons along the two dominant ascending somatosensory pathways [25,26]. Our investigation is motivated by the pioneering work of Ahissar and colleagues [27], who addressed the issue of pathways at the level of thalamus. These investigators made use of anesthetized animals, in which whisking was induced by electrical stimulation of the buccal motor branch of the facial nerve [28]. Under these conditions, the neuronal spikes rates are much reduced by the effects of anesthesia and the concurrent loss of neuromodulation. Furthermore, the process of electrical stimulation leads to the preferential activation of motoneurons with large caliper axons, as opposed to physiological recruitment, which begins with fibers of small caliper and progresses to those of larger caliper [29]. Thus there is a need for a thorough reexamination of the signaling of vibrissa input along ascending somatosensory pathways.

**Fig 1:**
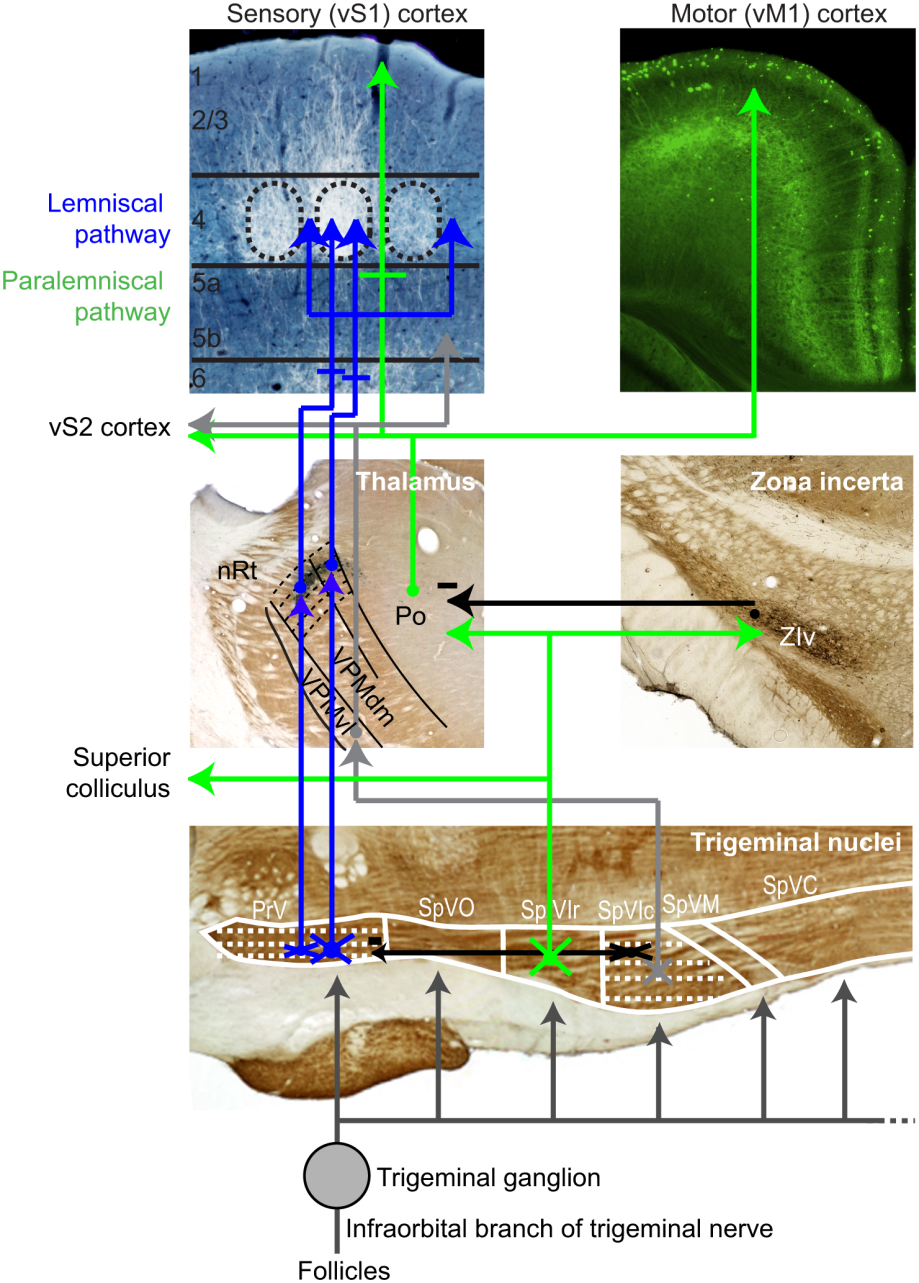
Map of vibrissa ascending pathways from the periphery to cortex. Dashed arrows represent individual barrelettes, barreloids, and barrels. The classical lemniscal pathway, including neurons with single- and multi-vibrissae receptive fields through the head and core of the barreloids, respectively, is shown in blue. The paralemniscal pathway is shown in green, and a third, extralemniscal pathway in grey. Inhibitory interactions are shown in black. Abbreviations: PrV, principal trigeminal nucleus; SpVIr and SpVIc, rostral and caudal divisions of spinal nucleus interpolaris, respectively; SpVM, spinal nucleus muralis; SpVC, spinal nucleus caudalis; VPMdm, dorsomedial aspect of the ventral posterior medial nucleus of dorsal thalamus; PO, medial division of the posterior group nucleus; nRt, nucleus reticularis; and ZIv, ventral aspect of the zona incerta. An original figure constructed with elements from Fig 3 in reference [19] and data from reference [41] to define the trigeminal borders.

The more familiar of the two pathways, the lemniscal somatosensory pathway, includes trigeminal nucleus principalis (PrV) and the upstream dorso-medial division of ventral-posterior-medial (VPMdm) thalamic nucleus (Fig 1). Neurons along this pathway spike vigorously in response to stimulus-induced deflection of one or multiple vibrissa [30–33]. Yet there is limited information on the nature of the response to vibrissa self-motion [34]. A second pathway, the paralemniscal pathway, encompasses the rostral aspect of spinal trigeminal nucleus interpolaris (SpVIr), the upstream posterior medial (PO) thalamic nucleus, and includes collaterals to the ventral aspect of zona incerta (ZIv), a region that further provides feedforward inhibition to PO thalamus (Fig 1) [35,36]. Neurons along this pathway in PO thalamus spike, albeit less prominently, in response to deflection of the vibrissae [31,37], yet there is apparently contradictory data on the nature of the self-motion response [27,38]. Lastly, we consider an alternate origin for whisking-related re-afference and ask if whisking is encoded by mechanoreceptors in the mystacial pad, which moves in phase with the vibrissae during whisking [39]. Encoding of self-motion in these receptors would represent re-afferent signals that are, in principle, independent of vibrissa touch. The result of these measurements defines the utilization of different pathways for sensorimotor signaling and constrains computational models of vibrissa-based object location [19,40].

## Results

### Assessment of Potential Proprioceptive Input in the Vibrissa System

**Fig 2:**
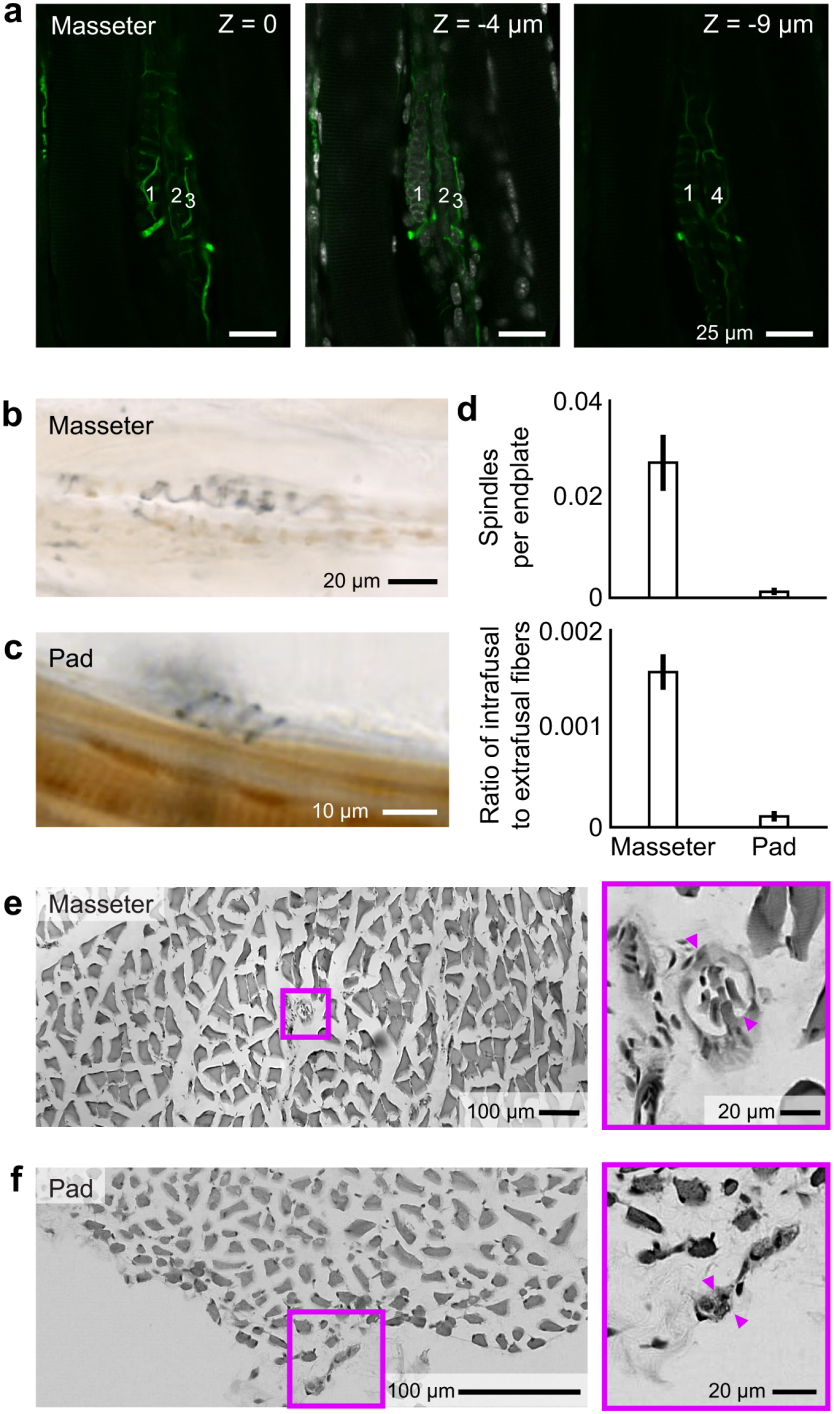
Identification of spindle complexes in the cranial muscles. **(a)** A spindle complex located in the masseter that is labeled by anti-Neurofilament H (green) with the nuclei in the underlying muscle labeled with DAPI (grey) at three imaging depths. Two nuclear bag fibers, labeled 1 and 4, and two chain fibers, labeled 2 and 3, form this complex. **(b,c)** Spindle sensory afferent fibers labeled by anti-Neurofilament H in the masseter (panel b) and the mystacial pad muscles (panel c). The muscle is stained with cytochrome oxidase (brown). **(d)** Fraction of labeled spindles relative to the number of labeled endplates in the same section, together with the fraction of observed intrafusal fibers per extrafusal fiber in the same section. **(e,f)**. Transverse sections of masseter (panel e) and mystacial pad (panel f), stained with hematoxylin and eosin stain to highlight all muscle fibers, together with higher magnification images that highlight intrafusal fibers (arrows). The raw data for panels b through f are in supplemental information S1 Data.

Although current evidence suggests a lack of proprioceptive innervation of most facial muscles in a number of species, data specific to the innervation of the rodent vibrissa musculature are more limited [8]. We therefore used three complementary anatomical techniques to determine whether vibrissa muscles contain endo-receptors (Fig 2).

First, a classic measure to observe endo-receptors is via the labeling of spindle-like proprioceptive afferent endings [42]. Spindles appear as helical-shaped fine processes that surround intrafusal muscle fibers. Spindles are well known to be prominent in the masseter muscle [43], as confirmed by immunostaining of neurofilament proteins from tangential sections of the muscle (Fig 2a). We thus searched for spindle-like endings in the mystacial pad, in both intrinsic and extrinsic muscles, as compared to sections of masseter muscle from the same animals. The number of motoneuron endplate claws in the same sections serves to normalize our counts. We observed spindles in the vibrissa musculature in only one of three animals (2,480 endplates across 23 sections) (Fig 2c), which correspond to 0.0012 ± 0.0007 (mean ± SD) spindles/plate compared to 0.0279 ± 0.0054 for the masseter muscle (970 endplates across 36 sections) (Fig 2b). Thus the vibrissa muscles contain over 20-fold fewer spindles than a muscle with known proprioceptive control (Fig 2d).

As a second measure, we prepared transverse sections of both the mystacial pad and the masseter muscles and directly stained both the intrafusal and extrafusal fibers. The intrafusal fibers are identified by their small size and bundling of multiple fibers within a capsule (arrows in Fig 2e and 2f). Here the total number of extrafusal fibers in a section serves as the normalization. We observed intrafusal fibers in the vibrissa musculature in two of three animals (53,800 extrafusal fibers across 13 sections) (Fig 2f), which corresponds 0.00011 ± 0.00005 intrafusal to extrafusal fibers compared to 0.00160 ± 0.00017 for the masseter muscle (56,960 extrafusal fibers across 13 sections) (Fig 2e). Thus the vibrissa muscles contain 15-fold fewer intrafusal fibers than a muscle with known proprioceptive control (Fig 2d).

As a final measure, we asked if γ-motoneurons, which innervate intrafusal fibers, are present in the lateral facial nucleus. This nucleus contains the motoneurons for the vibrissa musculature [44,45]. As a positive control, we compared staining in the lateral facial nucleus to the trigeminal motor nucleus, which innervates the masseter and other jaw muscles and, consistent with the presence of spindles in the masseter muscle (Fig 2a), is known to contain γ-motoneuron efferents [46,47]. Recently, it has been demonstrated that γ-motoneurons can be distinguished from α-motoneurons based on their size and the relative intensity of anti-ChAT and anti-NeuN staining. Specifically, both α- and γ-motoneurons are labeled intensely with anti-ChAT, but α-motoneurons have larger somata and are labeled by anti-NeuN, whereas γ-motoneurons are smaller and are not labeled by anti-NeuN [48].

**Fig 3:**
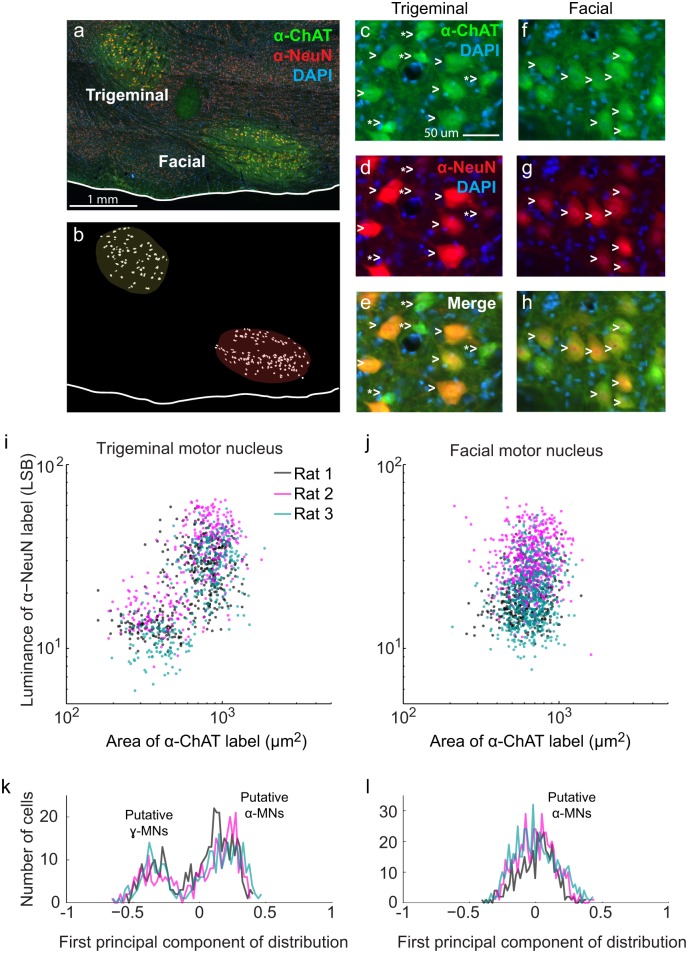
Identification of alpha and gamma motoneurons that innervate cranial muscles. **(a)** Rat brainstem section containing trigeminal and lateral facial motor nuclei. The section is labeled with anti-ChAT (green) anti-NeuN (red) and DAPI (blue). **(b)** Sample outlines of motoneurons in the trigeminal and lateral facial motor nuclei based on anti-ChAT labeling. **(c)** anti-ChAT labeling of motoneurons in the trigeminal motor nucleus. **(d)** anti-NeuN labeling of the neurons in **panel c**. **(e)** anti-ChAT and anti-NeuN labeling of the neurons in **panel c. (f)** anti-ChAT labeling of motoneurons in the facial motor nucleus. **(g)** anti-NeuN labeling of the neurons in **panel f**. **(h)** anti-ChAT and anti-NeuN labeling of the neurons in **panel f. (i)** Luminance of anti-NeuN label versus area of anti-ChAT label for trigeminal motoneurons in three rats. **(j)** Luminance of anti-NeuN label versus area of anti-ChAT label for facial motoneurons in three rats. **(k)** Histograms of the first principal component of log(Luminance) and log(Area) for the trigeminal motoneurons in **panel i. (l)** Histograms of the first principal component of log(Luminance) and log(Area) for the facial motoneurons in **panel j.** The raw data for panels i and j are in supplemental information S2 Data and that for panels k and l are in supplemental information S3 Data.

We analyzed immunohistochemical labeling on rat brainstem sections for ChAT and NeuN (Fig 3) and considered only neurons whose nucleus was contained in the section as indicated by a DAPI counterstain (Fig 3a and 3b). Qualitatively, the trigeminal motor nucleus contained two populations of motoneurons. Larger motoneurons were labeled both by anti-ChAT and anti-NeuN, whereas smaller motoneurons were labeled only by anti-ChAT (Fig 3c–3e). In the facial nucleus, we observed only one population of medium-sized motoneurons, presumably α-motoneurons, that were labeled both by anti-ChAT and anti-NeuN (Fig 3f–3h). To quantify these observations, we calculated the area of each motoneuron and the average intensity of anti-ChAT and anti-NeuN labeling within the labeled area. We observed two clusters of neurons in the trigeminal motor nucleus, putatively corresponding to α- and γ-motoneurons (Fig 3i and 3k). Approximately one-third of the motoneurons fell into the putative γ-motoneuron cluster, consistent with spinal motoneuron pools that innervate muscles with spindles [48]. In the facial motor nucleus we observed a unimodal distribution of motoneuron sizes and anti-NeuN intensities, putatively corresponding to α-motoneurons (Fig 3j and 3l). These results imply that the innervation of intrafusal fibers by the lateral facial motor nucleus represents at most a small fraction of its total output, and are consistent with past reports that most facial muscles lack proprioceptive signaling [7–11]. Together, these anatomical analyses of neuronal endings, muscle fibers, and motoneuron types imply that classic propriception makes a negligible contribution to the encoding of vibrissa self-motion.

### Spiking Activity in Trigeminal Brainstem Nuclei during Free Whisking

**Fig 4:**
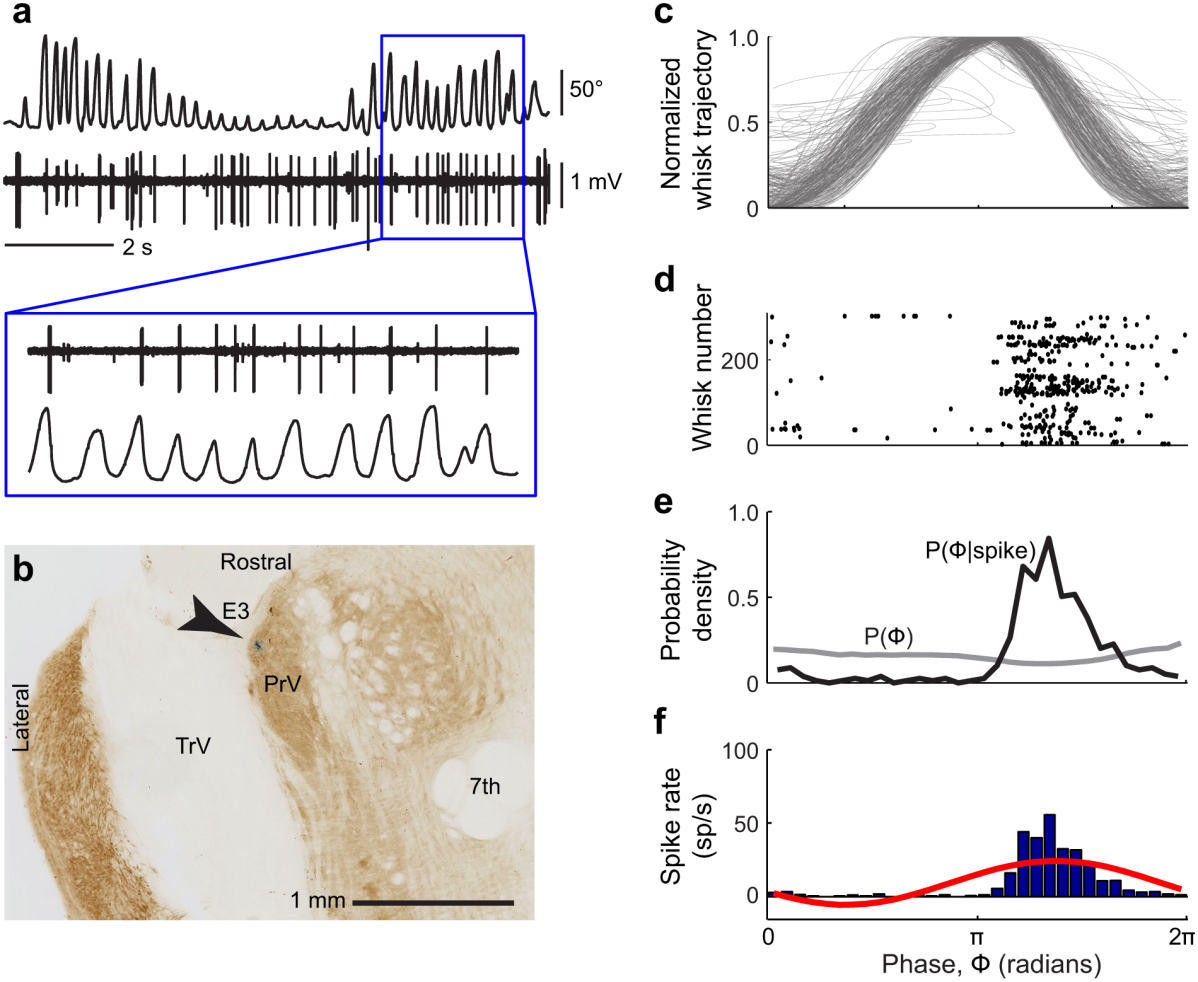
Spiking activity of a unit in nucleus PrV during free-air whisking. **(a)** Simultaneously recorded vibrissa motion and spiking activity during free-air whisking. **(b)** Location of the recorded unit in **panel a** in a horizontal brainstem section counterstained for cytochrome oxidase activity. Multi-unit activity at this recording site was detected in response to manual deflections of vibrissa E3. **(c)** Normalized vibrissa position as a function of phase in the whisk cycle, ϕ(τ). All whisks during the record are superimposed. **(d)** Raster of phases in the whisk cycle at which spikes occurred. Each line on the *y*-axis represents one whisk. **(e)** Probability density function of all observed instantaneous phases (gray) and phases conditioned on a spike (black). **(f)** Spike rate as a function of phase in the whisk cycle (blue) and a sinusoidal fit to the data (red).

Given the relatively poor proprioceptive innervation of the vibrissa musculature, re-afferent activation of trigeminal mechanosensory afferents, including lanceolate and Merkel ending neuron types, is a likely source of the sensory signal of whisking phase. We thus monitored neuronal activity in two of the target nuclei in the brainstem for these neuron types [15,16], nuclei PrV and SpVIr, that provide the majority of the ascending projections to thalamus (Fig 1). These nuclei anchor the lemniscal and paralemniscal pathways, respectively, and the literature is unequivocal about the presence of vibrissa touch responses in both nuclei. We recorded single and multi-unit activity in nucleus PrV (25 putative single unit and 31 multi-unit spiking signals) and nucleus SpVIr (14 putative single unit and 10 multi-unit spiking signals) (Methods). As illustrated by the example of Fig 4, the spike rates of units in nucleus PrV are substantially modulated on a cycle-by-cycle basis during rhythmic whisking in air (Fig 4a and 4b). To quantify this modulation of the spike rate, we isolated individual whisk cycles (Eq 1 and Eq 2) and aligned spike events relative to the instantaneous phase in the whisk cycle (Fig 4c,d) [49]. We next computed the distributions of whisking phases and of whisking phases at which spikes occurred (Fig 4e). From these distributions, we estimated the spike rate as a function of phase in the whisk cycle, (black line in Fig 4f) and fit a sinusoid rate function (Eq 4) to the data as a means to parameterize the modulation depth (Eq 5) and preferred phase. For the unit in Fig 4, the majority of spikes, i.e., 373/404 (92%) of spikes across 303 whisks, occurred during the retraction phase of the whisk cycle, when the vibrissae were moving in the caudal direction (Fig 4f).

**Fig 5:**
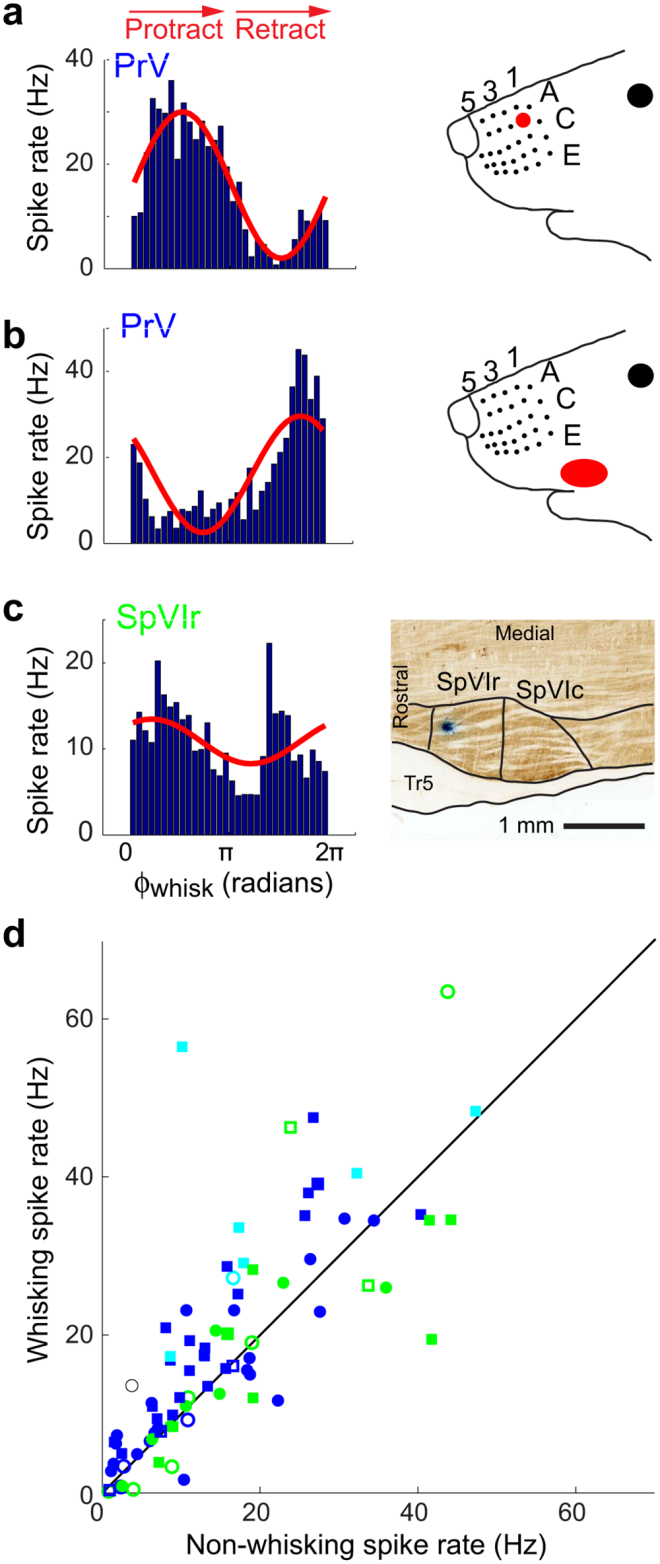
Spiking responses of units in nucleus PrV and SpVIr to free-air whisking. **(a)** Spike rate versus phase in the whisk cycle (blue histogram, left) and sinusoidal fit (red, left) of a unit in nucleus PrV. Multi-unit activity at the same recording site was elicited in response to deflections of vibrissa B1 (right). **(b)** Same as **panel a**, but for a unit in nucleus PrV that is located among units that responded to brushing the fur on the upper lip. **(c)** Spike rate versus phase in the whisk cycle (blue histogram, left) and sinusoidal fit (red, left) for a unit in nucleus SpVIr. Location of the recording site in a horizontal brainstem section (right). **(d)** Mean spike rates during whisking and non-whisking epochs for all putative single-unit (circles) and multi-unit (squares) recordings in nuclei SpVIr (green) and PrV (blue and cyan). PrV units that have macrovibrissa receptive fields (blue), as well as those that have facial skin, fur, or micro-vibrissa receptive fields (cyan) are shown. Of all of these trigeminal units, 49/56 units across three rats in nucleus PrV and 16/24 units across three rats in nucleus SpVIr were significantly modulated (Kuiper test, *p* < 0.05); solid symbols correspond to statistically significant modulation and open symbols to non-significant modulation. The raw data for panel d are in supplemental information S4 Data.

Tactile receptive fields were established for a subset of the recorded units by briefly anesthetizing the rat with isoflurane and manually stimulating different vibrissae (Methods). The unit in the example of Fig 4 was located among many units that responded to vibrissa E3. The firing rates of additional example units as a function of phase in the whisk cycle, along with their local receptive fields, are shown in Fig 5. These include units in the sub-region of nucleus PrV that corresponds to the macro-vibrissae (Fig 5a) and units in sub-regions that correspond to the skin and fur around the mouth and nose (Fig 5b). Furthermore, we observed units in nucleus SpVIr that were significantly, albeit modestly, modulated by whisking (Fig 5c). As a population, 49/56 PrV units (88%) and 16/24 SpVIr units (67%) were significantly modulated by whisking (Kuiper test, *p* < 0.05). Units in nucleus PrV tended to fire more spikes when the animal was whisking as opposed to not whisking (Wilcoxon signed rank test, *p* = 1.0 x 10^−5^), whereas spike rates were not significantly different between whisking and not whisking in nucleus SpVIr (Wilcoxon signed rank test, *p* = 0.39; Fig 5d).

**Fig 6:**
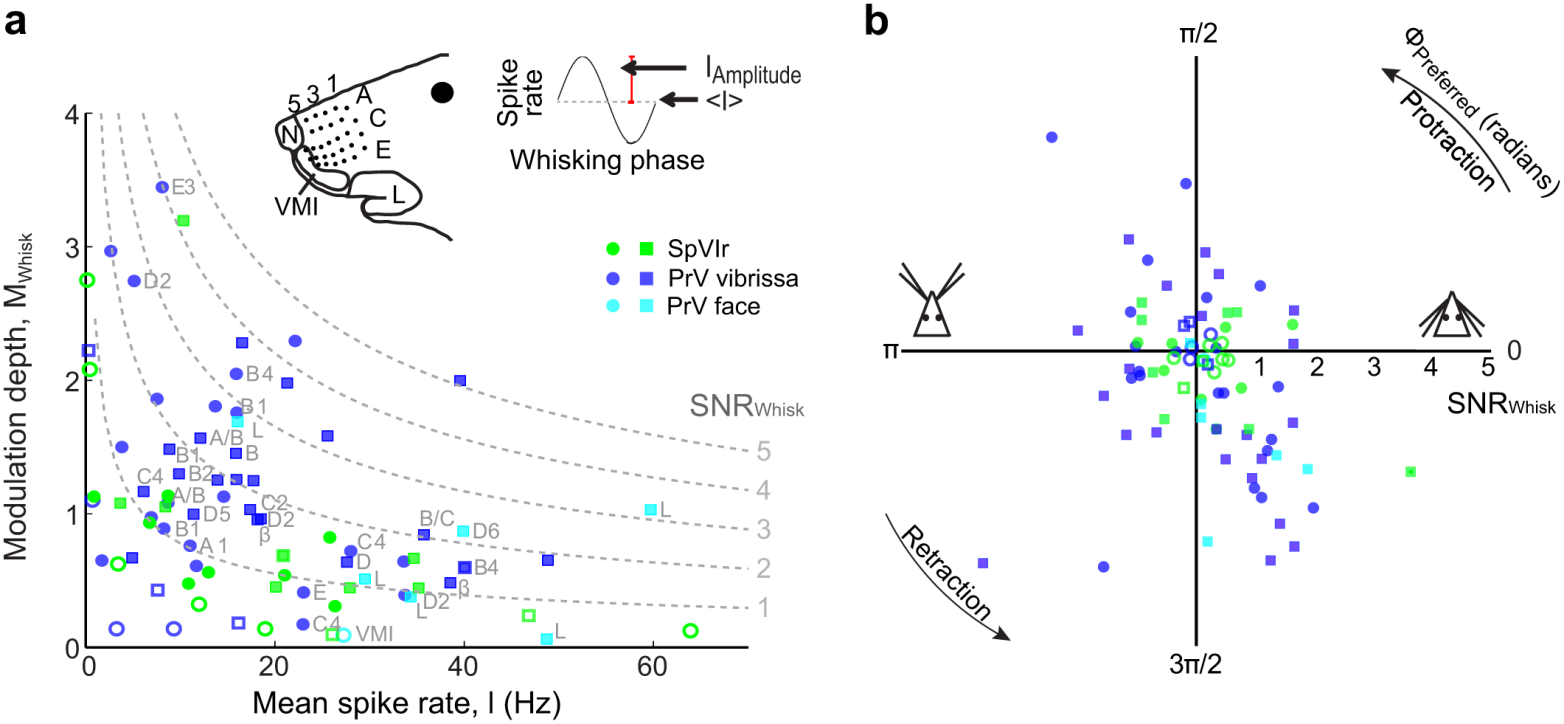
Modulation of spiking activity with free-air whisking in the trigeminal nuclei. **(a)** Whisking modulation, M_Whisk_, versus mean spike rate for all units, using the notation of Fig 5d. Contours with constant value of SNR_Whisk_ are shown as dashed lines for a temporal window of 165 ms. Approximate receptive fields at the recording sites are labeled. **(b)** Plot of the preferred phase (polar axis) versus SNR_Whisk_ (radial axis) for all trigeminal units. The raw data for panels a and b are in supplemental information S4 Data.

We characterized the sinusoidal fits of spike rates across all units (Figs 4f and 5a–5c) by two measures. The first measure is the modulation depth, M_Whisk_ (Eq 5), which reports the fraction of the unit’s response that is locked to whisking. The second measure is the signal-to-noise ratio, SNR_Whisk_, over a time interval (T) chosen to be the average period between whisks for head-fixed rats, i.e., T = 165 ms (Eq 6) [39,50]. We observe a greater modulation depth for lower mean spike rates, with a SNR_Whisk_ that peaks at a mean rate of <λ> ~20 Hz (Fig 6a). As a population, units in brainstem nucleus PrV were more strongly modulated than those in nucleus SpVIr (Wilcoxon ranked sum test, *p* = 0.03), with median M_Whisk_ values of 1.0 versus 0.6 for nucleus PrV versus SpVIr, respectively. Furthermore, units in nucleus PrV had a greater SNR_Whisk_ than those in nucleus SpVIr (Wilcoxon ranked sum test, *p* = 0.0016, with median values of SNR_Whisk_ = 1.6 versus 0.8 for nucleus PrV versus SpVIr, respectively.

Different units preferentially spiked at different phases of the whisk cycle, denoted ϕ_Preferred_ (Eq 4). All phases are represented for units in both nuclei PrV and SpVIr (Fig 6b). There is a significant bias in the preferred phase across all units in nucleus PrV, with a vector average <SNR_Whisk_> = 0.6 and <ϕ_Preferred_> = 4.9 radians (Hotelling’s one-sample test; *p* = 0.02); this phase corresponds to retraction from the fully retracted position. There was no bias for units in SpVIr (Hotelling’s one-sample test; *p* = 0.3) [51]. In toto, these data show that self-motion is represented along the primary nuclei of the lemniscal and paralemniscal pathways, but more robustly along the lemniscal pathway.

### Spiking Activity in Thalamic Nuclei and Zona Incerta during Whisking

**Fig 7:**
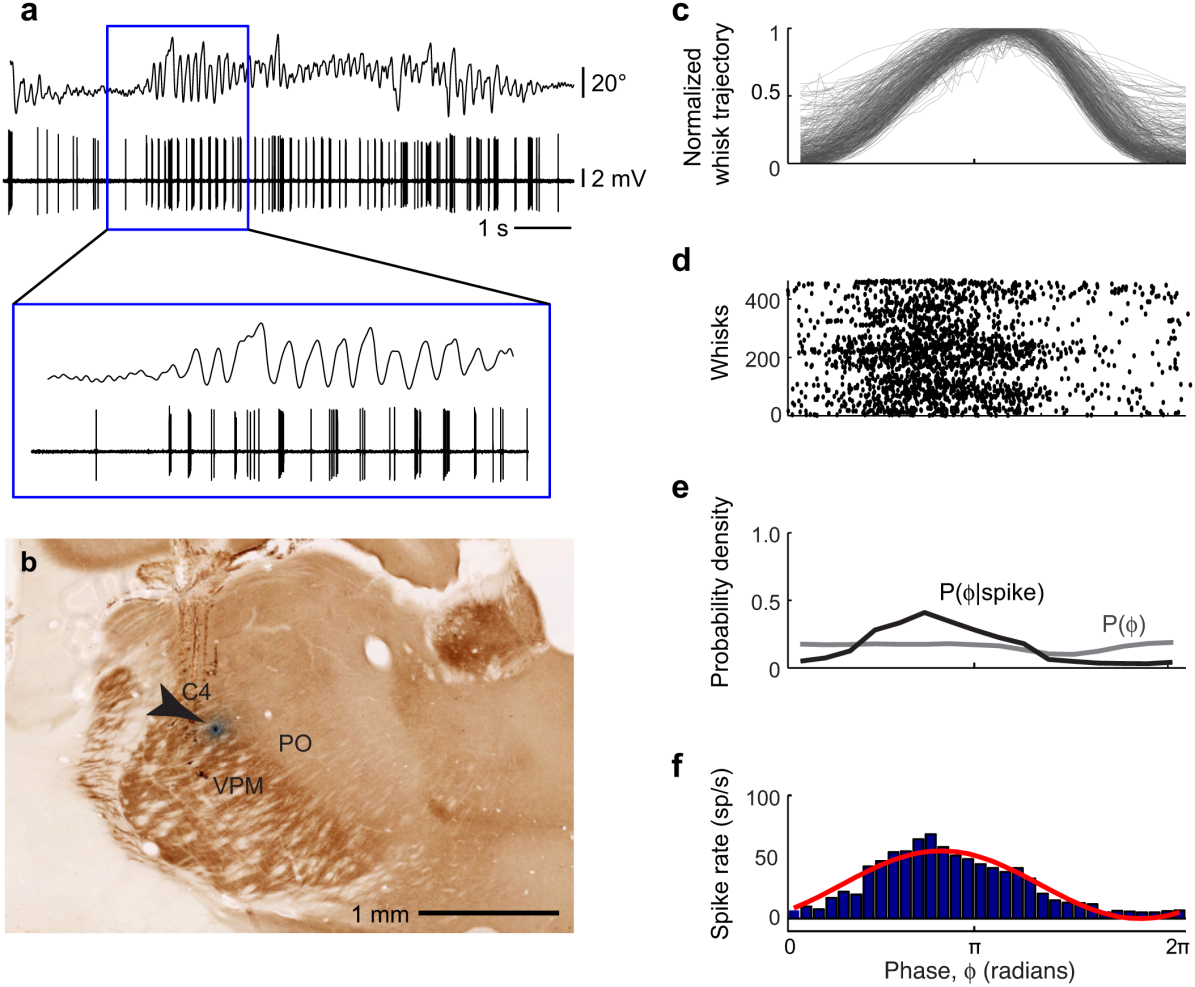
Spiking activity of a VPM neuron during free-air whisking. **(a)** Simultaneously recorded vibrissa motion and spiking activity during free-air whisking. **(b)** Location of the recorded unit in a coronal section. Multi-unit activity at this recording site was detected in response to manual deflections of vibrissa C4. **(c)** Normalized vibrissa position as a function of phase in the whisk cycle. All whisks during the record are superimposed. **(d)** Raster of phases in the whisk cycle at which spikes occurred. Each line on the *y*-axis represents one whisk. **(e)** Probability density function of all observed instantaneous phases (gray) and phases conditioned on a spike (black). **(f)** Spike rate as a function of phase in the whisk cycle (blue) and a sinusoidal fit to the data (red).

**Fig 8:**
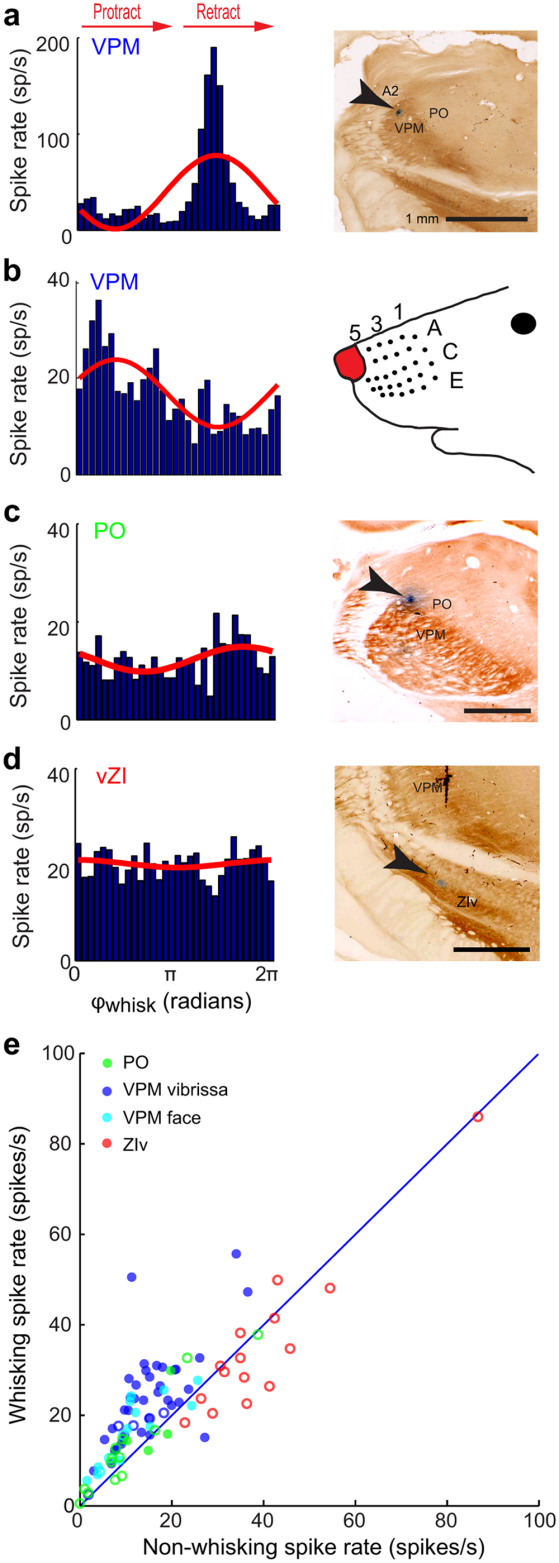
Spiking responses of additional VPM and PO thalamic and ZIv neurons to whisking in air. **(a)** Spike rate versus phase in the whisk cycle (blue histogram, left) and sinusoidal fit (red, right). Multi-unit activity at the same recording site was elicited in response to deflections vibrissa A2. The recording site was identified in VPM thalamus in post-hoc histology (right). **(b)** Spike rate versus phase in the whisk cycle and sinusoidal fit for a unit located in VPM thalamus. The unit was located among units that responded to brushing the naris (right). **(c)** Spike rate versus phase in the whisk cycle and sinusoidal fit for a unit located in PO thalamus. **(d)** Spike rate versus phase in the whisk cycle and sinusoidal fit for a unit located in ZIv. **(e)** Mean spike rates during whisking and non-whisking epochs for all VPM thalamic (blue and cyan), PO thalamic (green), and ZIv (red) neurons in this study. VPM neurons that have macrovibrissa receptive fields (blue), as well as those that have facial skin, fur, or micro-vibrissa receptive fields (cyan) are shown. Solid symbols correspond to statistically significant modulation and open symbols to non-significant modulation (Kuiper test, *p* < 0.05). The raw data for panel e are in supplemental information S5 Data.

**Fig 9:**
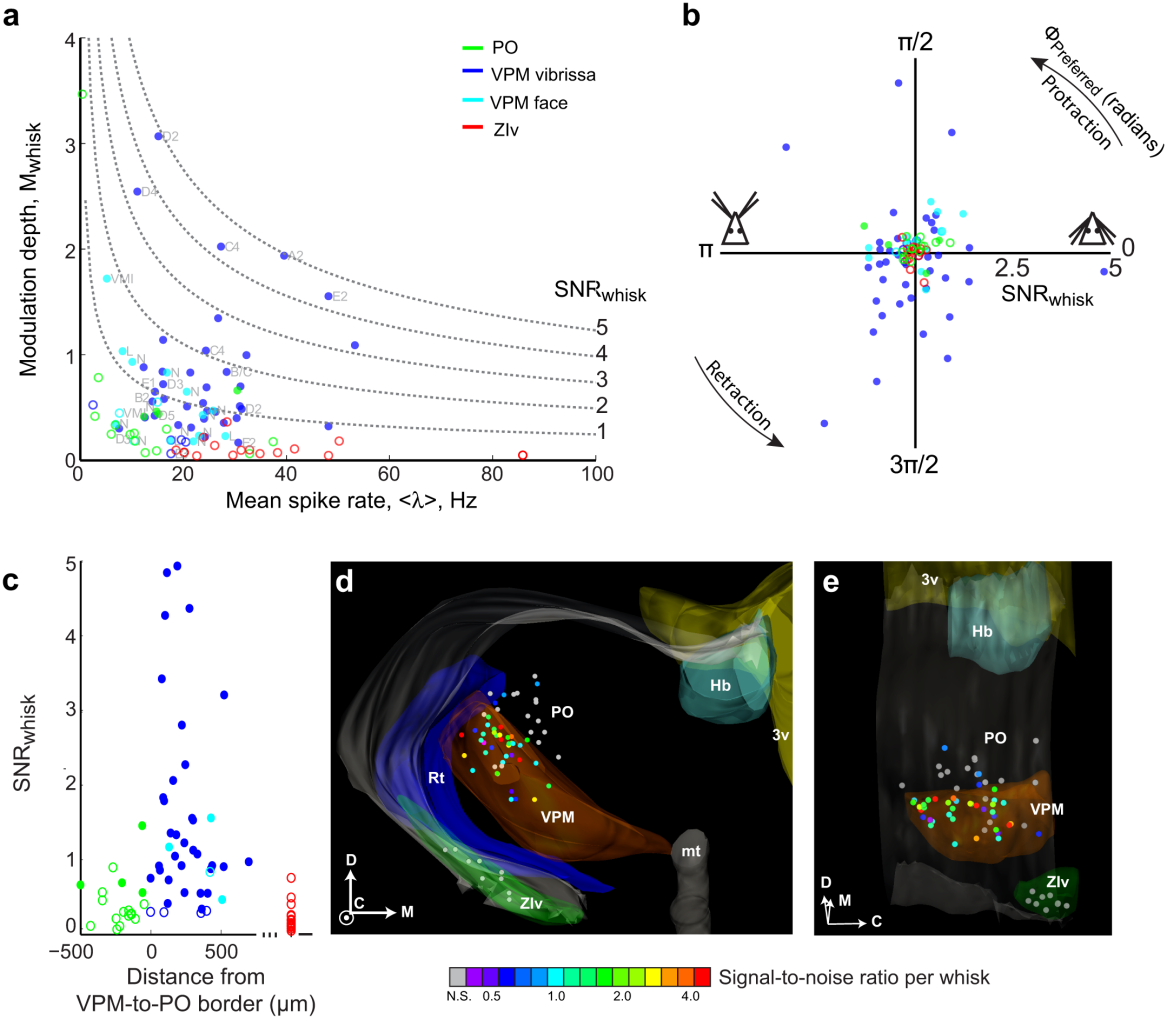
Compendium on modulation of spiking by free-air whisking by units in the thalamus and zona incerta. Modulation depth, M_Whisk_, versus mean spike rate for individual neurons in VPM, PO, and ZIv. Conventions are as in Fig 8e. Contours with constant values of SNR_Whisk_ are shown as dashed lines, and approximate receptive fields at the recording sites are labeled. **(b)** Plot of the preferred phase (polar axis) versus SNR_Whisk_ (radial axis) for the units in panel a. **(c)** The value of SNR_Whisk_ versus perpendicular distance to the VPM/PO border for the labeled VPM and PO recording sites in panel a. The values of SNR_Whisk_ for neurons in ZIv are shown at the right end (red). **(d)** Rostral view of the reconstructed locations of recording sites (circles) and select anatomical borders in three dimensions. The colors of the circles represent the SNR_Whisk_ for each recorded neuron; gray corresponds to neurons that were not significantly modulated by whisking phase. Labeled structures correspond to the third ventricle (3v, yellow), habenula (Hb, teal), mammothalamic tract (mt, gray), thalamic reticular nucleus (Rt, blue), and ventral division of zona incerta (ZIv, green). The anatomical axes are shown in the lower right hand corner: dorsal (D), medial (M), and caudal (C). **(e)** An oblique view of the reconstruction in panel d. The raw data for panels a through c are in S4 Data.

**Fig 10:**
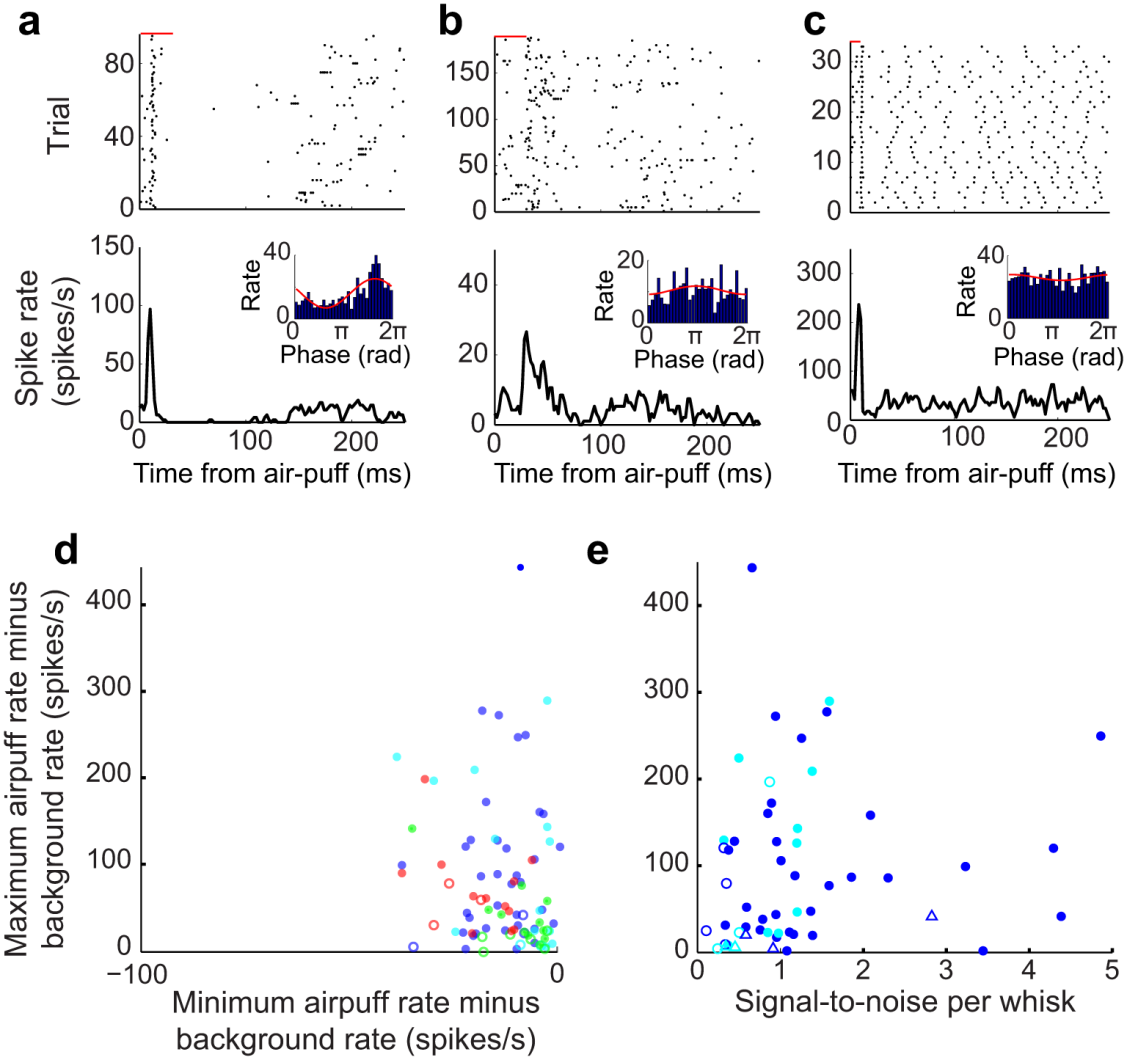
Modulation of spiking activity in response to vibrissa deflections induced by brief air-puffs for neurons in thalamus and zona incerta. **(a)** Raster of spike times relative to air-puff onset for a neuron in VPM thalamus (black ticks); the duration of the air-puff is shown by the red bar. A corresponding peri-stimulus time histogram (PSTH) is shown. The insert is the response of the same neuron to whisking in air, as in Fig 6a–6c. **(b)** Raster of spike times relative to air-puff onset for a neuron in PO thalamus. Conventions are as in panel a. **(c)** Raster of spike times relative to air-puff onset for a neuron in ZIv. Conventions are as in panel a. **(d)** Modulation of spike rates upon response to air-puff deflections for neurons in VPM thalamus (blue), PO thalamus (green) and ZIv (red) versus the baseline spike rate. Modulation is calculated both as the difference between the maximum spike rate in the first 100 ms in the PSTH **(panels a–c)** and the background rate (*y*-axis), and as the difference between the minimum spike rate and the background rate (*x*-axis). Units with both statistically significant (solid circles) and non-significant modulation (open circles) are shown (KS test, *p* < 0.05). **(e)** Modulation of spike rates upon air-puff calculated as the difference between the maximum and background rates, as in **panel d**, versus SNR_Whisk_ for VPM units. Solid circles represent units with statistically significant modulation during both air-puffs (KS test, *p* < 0.05) and whisking (Kuiper test, *p* < 0.05). Open circles represent units with statistically significant modulation during air-puffs only, and open triangles represent units with statistically significant modulation during whisking only. The raw data for panels d and e are in supplemental information S4 Data.

To determine the encoding of self-generated whisking in the thalamic nuclei that receive inputs from PrV and SpVIr (Fig 1), we recorded spiking activity of individual neurons using the juxtacellular configuration in VPM and PO thalamus (74 neurons). Occasionally, extracellular recordings of nearby units were obtained on the same micropipette; these units had negative initial deflections, as opposed to the initial positive spike deflections of the juxtacellularly recorded neurons (3 of 71 records). Similarly, we recorded spiking activity of individual neurons using the extracellular or juxtacellular configuration in ZIv (15 neurons). We next consider the spiking dynamics of individual neurons in VPM and PO thalamus, as well as in ZIv, in response to self-generated whisks (Figs 7–9) and external vibrissa deflections with air-puffs (Fig 10).

As illustrated by the example of Fig 7, in which the neuron was located among units that responded to vibrissa C4, neurons in VPM thalamus are substantially modulated on a cycle-by-cycle basis during whisking (Fig 7a and 7b). The analysis of the spike rate as a function of phase in the whisk cycle for thalamic neurons (Fig 7c–7f) proceeded similarly to that for units in the brainstem (Figs 4 and 5). For the neuron in Fig 7, the majority of spikes occurred during the protraction phase of the whisk cycle. The spike rate from this neuron was particularly well described by a sinusoidal modulation as a function of phase (Fig 7f).

Additional example neurons from VPM thalamus, the adjacent sub-region in PO thalamus, and ZIv, along with their anatomical locations of the recording sites, are shown in Fig 7, S1, S2 and S3 Figs. Qualitatively, neurons in the sub-region of VPM thalamus that corresponds to the macro-vibrissae (Fig 8a), as well as units in sub-regions that correspond to the skin or fur around the mouth and nose (Fig 8b), were modulated. PO thalamus also contained a minority of neurons that were modulated (Fig 7c), while modulation appeared absent in neurons in ZIv (Fig 8d). As a population, neurons in VPM and PO thalamus tended to fire more spikes when the animal was whisking as opposed to not whisking (Wilcoxon signed rank test, *p* = 10^−9^ and *p* = 0.04, respectively (Fig 8e). This is consistent with past results [52]. Neurons in ZIv tended to fire fewer spikes when the animal was whisking (Wilcoxon signed rank test, *p* = 0.02) (Fig 8e). Overall, neurons in VPM thalamus tended to have significantly higher spike rates than in those PO thalamus during whisking epochs (Wilcoxon ranked sum text, *p* = 0.0057), but not during non-whisking epochs (Wilcoxon ranked sum text, *p* = 0.15).

Similarly to the analysis for units in nuclei PrV and SpVIr, we characterized the population response for neurons in VPM and PO thalamus and ZIv in terms of the modulation depth, M_Whisk_ (Eq 5), and the signal-to-noise ratio, SNR_Whisk_ with T = 165 ms (Eq 6). The majority of neurons in VPM thalamus were significantly modulated by whisking phase (49/57; Kuiper test *p* < 0.05) (Fig 9a), whereas only a minority of PO neurons were significantly modulated (4/17; Kuiper test *p* < 0.05) (Fig 9a) and no neurons in ZIv were significantly modulated (0/15; Kuiper test *p* < 0.05). Of the VPM neurons located among units that had receptive fields corresponding to the micro-vibrissae or peri-mystacial fur, 12/15 of these neurons were significantly modulated. As in the case for brainstem (Fig 6b), different VPM neurons preferentially spiked at different phases of the whisk cycle. All phases are represented for neurons in VPM thalamus (Fig 9b), but with no significant bias in the preferred phase (Hotelling’s one-sample test; *p* = 0.72). In toto, these data show that self-motion is represented in thalamic nuclei of the lemniscal and paralemniscal pathways but, as with the case of brainstem, only robustly along the lemniscal pathway.

We computed the perpendicular distance between the Chicago sky blue spot and the VPM/PO border for each labeled recording site, based on cytochrome-oxidase stained sections. The location of the VPM/PO border, determined by visual inspection, was estimated to be accurate to approximately 80 μm (S4 Fig and S4 Data). There does not appear to be a clear systematic relationship between the signal-to-noise ratio for whisking and the distance to the border between VPM and PO thalamus at this spatial resolution. Neurons with high values of SNR_Whisk_ occur in VPM thalamus both close to the border as well as deeper in the nucleus (Fig 9c). To further clarify whether there is a potential segregation of function within VPM thalamus, we reconstructed the locations of the labelled recording sites in three dimensions (Fig 9d and 9e). Again, there is no clear spatial relationship between the location of a neuron within VPM and its SNR_Whisk_. The lack of topography would imply that self-generated motion and touch are signaled within the same anatomical pathway.

### Stimulus-Induced Spiking in Thalamic Nuclei and Zona Incerta

To determine whether the same neurons respond to ex-afferent and re-afferent stimuli, we next consider how the same neurons along the lemniscal and paralemniscal pathways respond to external deflections of the vibrissae. The case for touch-based responses in the VPM thalamus, along the lemniscal pathway, is unequivocal. However, the case for touch-based responses in PO thalamus, along the paralemniscal pathway, is the subject of conflicting reports as to whether external stimuli can drive neurons in PO thalamus independent of feedback activation from deep layers in cortex. As past work involved anesthetized animals [37,38,53–55], we undertook a re-analysis of the response of neurons in VPM and PO thalamus along with the somatosensory region of ZIv (Fig 1).

As illustrated by the examples of Fig 10a–10c, neurons in all three areas were modulated by air-puff deflections to multiple vibrissae and peri-mystacial fur, with neurons in VPM thalamus responding vigorously, those in PO thalamus the least responsive (Fig 10b), and those in ZIv responding with short latency, precisely timed spikes (Fig 10c). Across the population, 49/54 VPM neurons (91%), 11/17 PO neurons (65%), and 12/15 (80%) ZIv neurons were significantly modulated by air-puffs (*p* < 0.05) (Fig 10d). These data imply that nucleus SpVIr indeed drives ascending targets and that neurons in PO thalamus are responsive to stimulation in alert rats.

We next consider the responses of these same neurons to self-motion of the vibrissae (inserts in Fig 10a–10c). Consistent with the notion of a single anatomical pathway for re-afferent whisking and ex-afferent touch, the majority of VPM units that were modulated by self-generated whisking tended to also be modulated by air-puff deflections. Of the neurons in VPM thalamus, 42/54 (78%) were significantly modulated by both air-puffs and whisking, five were modulated by whisking only, and seven were modulated by air-puffs only. Yet there does not appear to be a relationship between the fidelity of modulation for VPM neurons that are significantly modulated by both whisking and air-puffs, as measured by the correlation between signal-to-noise ratio for whisking and the peak modulation upon air-puff (Fig 10e), (r = 0.05 with *p* = 0.76 for VPM units).

## Discussion

We report the representation of self-generated whisking in subcortical somatosensory brain regions (Fig 1). First, we assess the potential contribution of proprioceptive endings in the facial musculature to somatosensation. We find a small number of previously unreported spindle-like endings and intrafusal fibers within the vibrissa musculature. However, these endings fibers are relatively scarce in comparison with the nearby masseter muscle (Fig 2). Furthermore, using a recently developed immunohistochemical strategy [48], we find that the lateral facial motor nucleus contains few, if any, intrafusal fiber-innervating γ-motoneurons (Fig 3). While we cannot rule out the possibility that intrafusal fibers are instead innervated by the hypoglossal [56] and trigeminal mesencephalic nuclei [57], this finding, together with comparatively low density of spindles and intrafusal fibers, would suggest that vibrissa position is unlikely to be sensed by proprioception [58]. Nonetheless, we find a robust representation of self-generated vibrissa motion, i.e. whisking, in nucleus PrV (Fig 4), which receives primary afferent input from mechanoreceptors in the vibrissa follicles and the face. These results, together with previous findings that the representation of rhythmic vibrissa motion in somatosensory afferents are derived from peripheral sensors [14,59,60], leads us to conclude that vibrissa position during whisking is encoded through re-afferent activation of mechanical exo-receptors (Figs 4–6).

We next established the modulation of spiking activity of neurons in the lemniscal and paralemniscal pathways by the phase in the whisk cycle. At the level of the trigeminal brainstem, lemniscal neurons in nucleus PrV are substantially more reliable encoders of phase than paralemniscal neurons in spinal trigeminal nucleus SpVIr (Figs 5 and 6). At the level of the thalamus, lemniscal neurons in VPM thalamus are again substantially more reliable encoders of phase than paralemniscal neurons in PO thalamus (Figs 7–9). In particular, the majority of PO thalamic neurons do not significantly encode phase (Figs 7–9). Consistent with the lack of whisking-related modulation in PO thalamus, neurons in ZIv, which receive inputs from axon collaterals of cells in SpVIr that primarily project to PO-thalamus (Fig 1), are also not modulated by whisking (Figs 8 and 9). Together these data indicate that the lemniscal pathway from brainstem to cortex contains both neurons with the highest acuity for passive vibrissa deflections and neurons with the greatest reliability for encoding phase in the whisk cycle. Some single units are reliable encoders of both signals (Fig 10a and 10e), as proposed by studies that utilized electrically induced whisking in anesthetized animals [27,61].

### The Lemniscal Pathway in Encoding Self-Motion and Touch

Whisking-phase responses observed in VPM thalamic neurons in the present study substantially extend the results of past studies performed with both alert [34] and anesthetized [27,61] rats. We observe phase-dependent spiking modulation throughout the depth of VPMdm thalamus, which presumably comprises units in both the “head” and “core” regions of the barreloids [62]. This finding is consistent with results in which artificial whisking was induced by electrical stimulation of the facial nerve in anesthetized rats [27]; however, we find that units are tuned to all phases of the whisk cycle rather than to protraction onset. These broader distributions of preferred phases, which are observed in both PrV and VPM thalamus (Figs 6b and 9b), are consistent with the range of phase preferences observed somatosensory cortex during natural whisking [12,13,20,63,64]. We were unable to assess whether there is a finer systematic map of the encoding of self-motion on the scale of individual barreloids [62,65]. Interestingly, in addition to the barreloids, we observe modulation in phase with whisking in some units that encode distortions to the skin or fur outside of the vibrissa follicle in both PrV and VPM thalamus (Figs 5b, 6a, 8b, 8e and 9).

**Fig 11:**
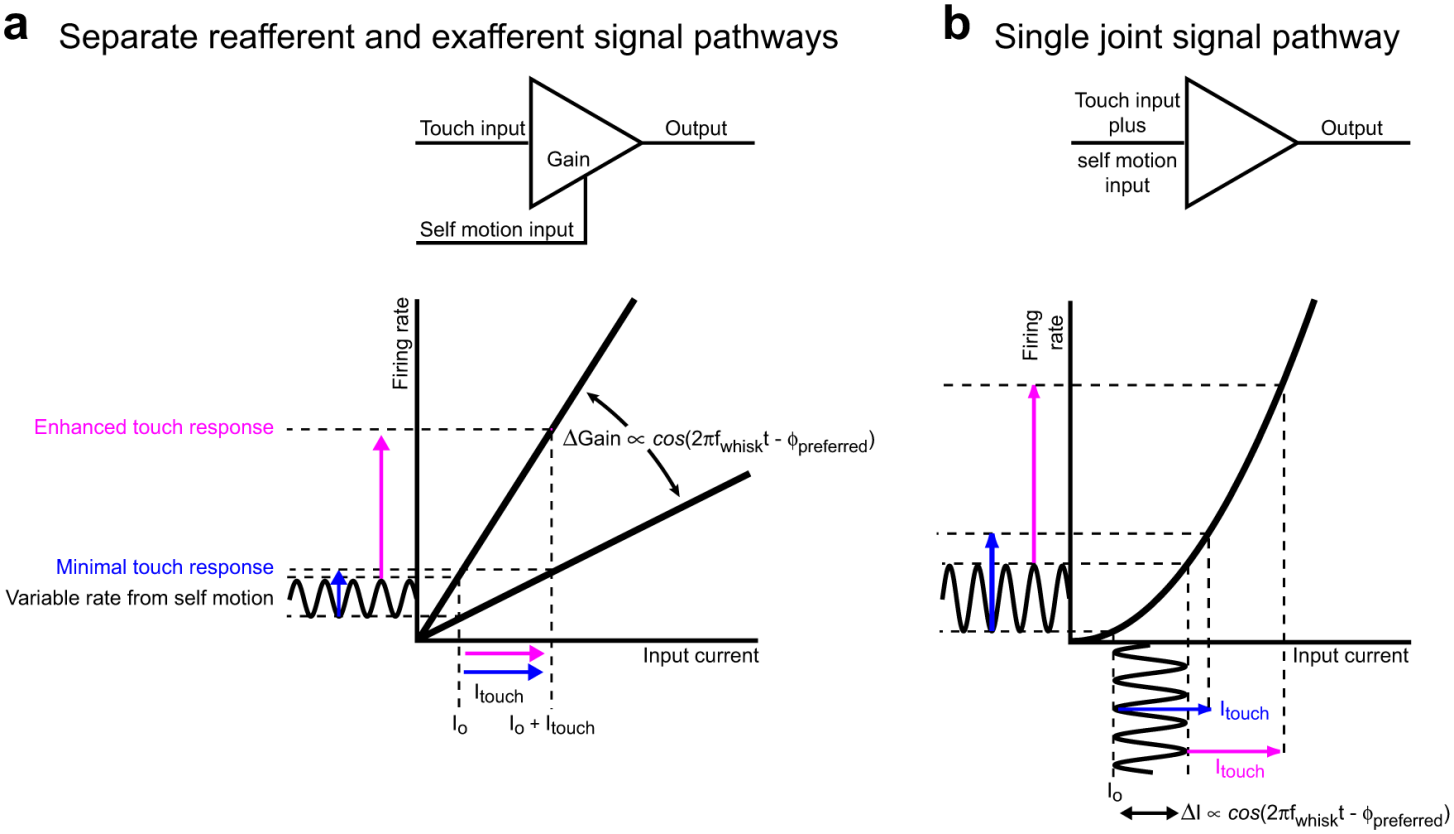
Schemes for demodulation of touch signals by vibrissa whisking signals. **(a)** A scheme for a parallel pathway for the ex-afferent signal and a reafferent signal that encodes phase in the whisk cycle independent of touch to the vibrissae. The slope of the f-I curve is assumed to be modulated by the reafferent signal, as can occur if re-afference drives shunting inhibition. This scheme is relevant if the encoding of whisking by the skin and fur serves as the reafferent signal. **(b)** A scheme for a single pathway for both the ex-afferent signal, which encodes touch to the vibrissa, and re-afferent signal, which encodes phase in the whisk cycle, measured in terms of free whisking in air. The signals are mixed by a spike rate versus input current (f-I) function that has an accelerating slope. This scheme is relevant if both the ex-afferent and re-afferent input share the same ascending lemniscal input from VPM to layer 4 cortical neurons.

The observation that the majority of whisking responses are encoded within the lemniscal pathway raises the question of how phase-dependent touch signals, which were previously observed in somatosensory cortex [20], arise from the observed thalamic inputs. There are at least two potential schemes that could produce these cortical phase-dependent touch signals (Fig 11). One scheme is that whisking and touch are encoded by different populations of peripheral mechanoreceptors and central neurons. In this scheme, thalamic neurons that predominantly encode the whisking signal could change the slope of the gain function of cortical neurons, i.e., the proportionality of spike rate to input current [20], in a phase-dependent manner (Fig 11a), analogous to heterodyne detection [23]. Contrary to previously proposed hypotheses [20,27], our data indicate that paralemniscal inputs are unlikely to be the source of this cortical gain modulation. However, lemniscal units that encode skin or fur distortions during whisking, which we observed in nucleus PrV and VPM thalamus, could in principle contribute to a re-afferent signal of vibrissa position that is independent of vibrissa touch (Figs 5b and 8b). It remains to be determined whether such signals can influence phase-dependent touch responses in the barrels of somatosensory cortex.

A more parsimonious scheme is that the same mechanoreceptors, PrV neurons, and VPM thalamic neurons encode both whisking and touch signals. In this scheme, a gain function with an accelerating nonlinearity [24] could enhance the spike rate at the peak of the whisking signal relative to other positions (Fig 11b), in analogy to homodyne detection [23] and the effect of a threshold nonlinearity [66]. Based on the present results, units that encode both whisking and external vibrissa deflections could provide the relevant inputs to somatosensory cortex (Fig 10a and 10e). According to this scheme, if touch occurs at preferred phase of the whisk cycle, the response is enhanced, while touch at the non-preferred phase leads to a diminished response. Such non-linear gain functions could be present at multiple stages along the sensory processing stream, including at the mechanoreceptors themselves. In fact, modulation of touch by self-motion can occur even if self-motion signal alone is sub-threshold, and the resulting threshold nonlinearity can further enhance the difference between touches at different phases.

### Is There a Role for the Paralemniscal Pathway in Encoding Self-Motion and Touch?

The potential role of the paralemniscal pathway in sensing vibrissa motion is controversial [27,38,53,67–70]. The majority of neurons in nucleus SpVIr are similarly tuned to upward vibrissa deflections of many vibrissae in anesthetized rats [71], but are only weakly tuned to phase during whisking relative to neurons in nucleus PrV (Figs 5 and 6). Neurons in PO thalamus respond only weakly to external vibrissa deflections as a consequence of feed-forward inhibition from the output of ZIv neurons in ketamine-anesthetized rats [36]. Electrical stimulation of vibrissa motor cortex inhibits activity in ZIv, which disinhibits neurons in PO thalamus and thereby increases its responsiveness to deflections [69]. This observation led to the hypothesis that whisking-related activity in primary motor cortex [49,72] might gate PO thalamus so that it is sensitive to whisking. Our data suggest that while the overall firing rates of ZIv neurons decrease slightly during whisking, this decrease is not sufficient to elicit whisking-phase dependent responses in PO thalamus. The lack of phase-dependent responses in the majority of PO units in our study is consistent with a past report [38] but inconsistent with results obtained with electrically induced whisking in urethane-anesthetized rats [27]. Nonetheless, it is interesting that PO thalamic neurons have been shown to respond to vibrissa movements in the latter condition. In this respect, it remains possible that PO thalamic neurons are able to respond in a similar manner to SpVIr during a currently unknown behavioral context.

### Why Encode Self-Motion through Peripheral Reafference?

In the absence of proprioception (Figs 2 and 3) and corollary discharge [12], encoding of self-generated vibrissa movement through re-afferent activation of mechanoreceptors is a means for the animal to compute the position of its vibrissae [17]. This can be used to modulate the sensory response to touch depending on phase in the whisk cycle (Fig 11). Why does self-generated movement appear to be represented differently in the vibrissa system than in the limbs, where proprioceptive and cutaneous signals are encoded in separate thalamocortical pathways [73,74]? One possible explanation is that the limbs, which support the body, are likely to carry a variable load. Accurate positioning therefore requires sensory information related to muscle length and force that is independent of tactile sensation. This may also be true for jaw muscles, which are innervated by muscle spindle fibers [43] and corresponding γ-motoneurons (Fig 3) [46,75]. The vibrissa muscles, on the other hand, support only a small, relatively constant load that consists solely of the vibrissae, which readily flex upon the application of external forces [76,77]. While proprioception appears to exist in the extraocular muscles [78,79], other facial muscles that carry a small, relatively constant load are thought to be devoid of proprioceptive innervation [7–11]. We can only conjecture that facial expression control may follow similar mechanisms. In the case of other facial movements in which self-motion is encoded by exo- as opposed to endo-receptors, any position-dependent signal may serve as a reference signal for computing sensation in terms of sensor position.

## Methods

This study was performed in strict accordance with the recommendations in the Guide for the Care and Use of Laboratory Animals of the National Institutes of Health. The protocol was approved by the Committee on the Ethics of Animal Experiments of the University of California at San Diego (Protocol numbers: S02173 and S02174R).

### Animals

Fifty-four female Long Evans rats, 250 to 350 g in mass (Charles River), were used for combined anatomical, behavioral, and electrophysiological experiments. All behavior and electrophysiological data were obtained from head-restrained rats [80,81].

### Staining for Spindles and Fibers

Rats were transcardially perfused with 0.1 M phosphate buffered saline (PBS) followed by 4% (w/v) paraformaldehyde in PBS. Whole rat heads were post fixed for 4 to 12 h at 4°C. Muscles were dissected off of the fixed heads and cryoprotected in 30% (w/v) sucrose in PBS for 8 to 12 h at 4°C.

Both mystacial pad and masseter muscles were sectioned tangentially at a thickness of 60 μm with a sliding microtome. Sections were incubated in 2% (w/v) goat serum (S-1000, Vector) block for 30 min and then the primary antibody rabbit anti Neurofilament H 1:500 (Ab 1991, Millipore) overnight at room temperature. For fluorescent staining, secondary antibodies raised in goat were used (rabbit anti-594, A-11012, Life technologies). For dark product staining, sections were incubated in biotinylated rabbit secondary antibody (BA-1000, Vector) for 90 min followed by processing with an ABC kit (PK-6100, Vector) and the SG peroxidase kit (SK-4705, Vector). Sections were either initially counterstained with cytochrome oxidase or a solution of 0.25% (w/v) Eosin Y in 79% ethanol and 21% water.

Mystacial pad and masseter muscles were frozen in blocks of OCT (25608–930, Tissue-Tek) and sectioned transversely at a thickness of 10 μm with a cryostat. Sections were directly mounted on slides to maintain the integrity and orientation of the muscle fibers. They were left to dry for a minimum of 1 h. Slides were rehydrated and, sequentially, incubated in Mayer’s Hematoxylin Solution (MHS15-500, Sigma-Aldrich) for 8 min, washed with running tap water for 5 min, differentiated in a 1% (v/v) hydrochloric acid in distilled water for 30 s, further washed with running tap water for 2 min, “blued” in a saturated lithium carbonate solution (1.4% [w/v] lithium carbonate in distilled water) for 30 to 60 s, washed for 5 min in running tap water, rinsed by dipping 5 to 7 times in 95% (v/v) ethanol in water, counterstained with a 0.25% (w/v) Eosin Y solution in 79% ethanol and 21% tap water for 2 min, finally dried in air, and cover slipped using mounting media (06522, Sigma Aldrich).

Confocal stacks of images of spindle fibers were obtained with a Leica Sp5. Dark product, hematoyxlin, and eosin stained slides were imaged with a slide scanning microscope (Nanozoomer 2.0 HT, Hamamatsu). Fibers were counted using Photoshop (CS4, Adobe).

### Staining for Motoneurons

Rats were perfused and fixed and the brains were extracted and sectioned at a thickness of 30 μm, as above. Sections containing trigeminal and facial motor nuclei were incubated overnight in anti-ChAT (1:100 AB144P, Millipore) and anti-NeuN (either 1:100 MAB377, Millipore, or 10 μg/mL of a custom anti-NeuN directly conjugated to Alexa 594(Chemicon [82]). Sections were then rinsed and incubated for 90 min in anti-goat Alexa 488 (1:200 A11055, Invitrogen) and anti-mouse Alexa 647 (1:200 A31571, Invitrogen), rinsed again, mounted, and coverslipped.

Slides were scanned as described above. Motoneurons in the trigeminal and facial motor nuclei that contained a DAPI-stained nucleus were manually outlined based only on the anti-ChAT label (green channel) using Neurolucida software. The area and average intensity of the anti-NeuN label (red channel) within the outlined perimeter was then calculated.

### Recording

#### Brainstem

We recorded single- and multi-unit neuronal signaling in the trigeminal complex of the head-fixed awake and alert rat using quartz micropipets as extracellular electrodes. A craniotomy was made based on stereotaxic coordinates, centered at 9 mm posterior and 3 mm lateral to Bregma for PrV and 12 mm posterior/3 mm lateral to Bregma for SpVir. Trigeminal PrV and SpVIr nuclei were identified based on their stereotaxic coordinates and their multi-unit spiking responses to deflections of the macro-vibrissae, micro-vibrissae, and peri-mystacial fur with the use of 20 to 50 ms air-puffs delivered at 0.1 to 0.2 Hz. As the position of the vibrissae were continually shifting in the awake animal, we used broad puffs that essentially stimulated all of the vibrissae [83]. After a set of recordings were complete, we briefly anesthetized the rat with isoflurane, while maintaining the micropipet in the same location, so that the topology of the receptive field could be mapped. We manually deflected small patches, i.e., approximately 1 to 5 mm, of peri-mystacial fur as well as individual macro- and micro-vibrissae with a handheld probe and listened for spiking activity on an audio monitor. Lastly, we marked the location of the neighborhood of units by iontophoretic injections of Chicago sky blue dye and determined the anatomical location in post-hoc histology [50]. Animals underwent 1–3 d of recording, in which 3–14 units were recorded per day. We used previously described post-hoc spike sorting procedures [84,85].

#### Thalamus

We used neuronal recording procedures similar to those reported for brainstem recordings to examine spiking in head-fixed alert rats within VPM and PO thalamus and ZIv, except that recordings were made in the juxtacellular configuration [86,87] using micropipettes with 1 to 3 μm tip diameters. Briefly, a craniotomy was made based on stereotaxic coordinates, centered 3 mm posterior and 3 mm lateral to Bregma. We first identified these regions based on stereotaxic coordinates and spiking responses to broad air-puffs that simultaneously deflected the macro-vibrissae, micro-vibrissae, and peri-mystacial fur. Air-puffs were 5 to 20 ms delivered at 1 to 3 Hz. The somatosensory region of ZIv is located ventrally to the portion of VPM that corresponds to the A and B rows of vibrissae. We identified units in PO thalamus based on a 400 μm proximity to the border to the functionally identified VPM region. Once a unit was located we continued to move the micropipette in 1 μm increments until the spike waveform displayed an initial positive voltage deflection, which was usually accompanied by a 1.5-times or greater increase in resistance of the pipet. Recordings sites were labeled, as above. After a subset of the VPM thalamic recordings, we anesthetized the rat with isoflurane to determine the approximate receptive field of the recorded unit, as described above.

#### Anatomy

Recording sites were localized by concurrent dye injections from the recording pipet in all animals. Animals were perfused and fixed and the brains were extracted and sectioned at 60 μm thick [50]. Sections were stained for cytochrome oxidase reactivity [88] and scanned at a resolution of 0.5 μm/pixel on a slide scanner (Nanozoomer, Hamamatsu). Standard anatomical features were traced for the sections that contained the dye labels with the use of Illustrator software (Adobe). A reference atlas was prepared from a single, un-injected brain that was sectioned and prepared in the same manner.

Sections that contained a dye label were manually assigned to the nearest plane in the reference atlas. The sections were then manually rotated and scaled to approximately match the corresponding anatomical borders and standard anatomical features in the reference atlas. A subset of the following structures were used for alignment: ventrobasal thalamus, VPM thalamus, VPL thalamus, thalamic reticular nucleus, habenula, dorsal lateral geniculate nucleus, ventral lateral geniculate nucleus, dorsal zona incerta, ventral zona incerta, subthalamic nucleus, external medullary lamina, mammilothalamic tract, fornix bundle, and the third ventricle. The mapped locations of the recording sites on the reference atlas, along with nearby anatomical borders, were traced and reconstructed in three dimensions using Neurolucida software (Microbrightfield).

### Behavior

Vibrissae were clipped to approximately 2/3 of their original length and vibrissa position was monitored simultaneously with neuronal spiking activity under two behavioral conditions. First, as the rats were coaxed to whisk in air by presenting food or bedding from their home cage [83,89]. Second, as vibrissae were deflected externally by brief puffs of air applied to the face [81,83]. We monitored vibrissa position with a Basler A602f camera and a white light emitting diode backlight [50]. We chose a spatial resolution of 120 μm/pixel, a field of 360 × 250 pixels, a frame rate of 250 Hz, and a trial time of 10 s. The pixel intensity in the image was thresholded and the mean position of the full set of vibrissae was tracked by computing the center of mass of the thresholded pixels in each frame. The data were then converted into whisking angle versus time, denoted θ(t). Lastly, a Hilbert transform was used to decompose the whisking angle, θ(t), into the phase within the whisk cycle, ϕ(t), with

$$\theta \left(t\right)=\theta_{\text{Amplitude}}\text{cos}\left[\phi \left(t\right)\right]\;+\;\theta_{\text{Midpoint}}$$ (1)

where θ_Amplitude_ and θ_Midpoint_ are slowly varying parameters and the whisking frequency, f_whisk_, is given by [49]:

$$f_{Whisk}=\frac{1}{2\pi }\;\frac{d\phi \left(t\right)}{dt}.$$ (2)

Lastly, we recall that the vibrissae tend to move in phase with one another during free-air whisking [49]; thus the phase, but not the amplitude or midpoint, of all vibrissae may be taken as identical.

### Analysis

#### Free-whisking

The range of possible phases, *i*.*e*., 0 to 2π radians, was divided into 32 bins, each spanning π/16 radians. Thus phase is now labeled by an index k with 1 ≤ k ≤ 32. Each spike that occurred during a detected whisk, at time t_S_, was assigned an instantaneous phase ϕ(t_S_) and thus assigned to one of the phase bins. We define N(ϕ_k_|spike) as the number of spikes in phase bin k across all detected whisks and P(ϕ_k_|spike) = N(ϕ_k_|spike)/N_spikes_, where N_spikes_ is the total number of spikes, as the probability density function of phase for spiking events. Since the distribution of phases is not uniform across time, we similarly computed the number of phases observed for each bin, denoted N(ϕ_k_), along with the probability density function of the number of whisking events for each phase bin, denoted P(ϕ_k_). These two distributions used to compute the mean spike rate for each phase bin, ϕ_k_, which we denote λ[ϕ_k_] and define as:

$$\lambda \left[\phi_{k}\right]=r\cdot \frac{N(\phi_{k}|spike)}{N\left(\phi_{k}\right)}$$ (3)

where *r* is the video frame rate.

We characterized the modulation of each unit by fitting a sine wave with a period 2π to the rate versus phase in the whisk cycle with standard linear least-squares regression techniques. The fit, defined by:

$$\hat{\lambda }\left(t\right)=\;\langle \lambda \rangle +\lambda_{Amplitude}\text{cos}\left[\phi \left(t\right)-\phi_{Preferred}\right],$$ (4)

is completely described by its mean, <λ>, amplitude, λ_Amplitude_, and phase, ϕ(t). A preferred phase of ϕ_Preferred_ = 0 corresponds to fully retracted while ϕ_Preferred_ = ± π corresponds to fully protracted.

The modulation depth of the averaged whisking response is defined as:

$$M_{Whisk}\;\equiv 2\frac{\lambda_{Amplitude}}{\langle \lambda \rangle }$$ (5)

and the signal-to-noise ratio for a point process with Poisson distributed arrival times and a temporal window of T, denoted SNR_Whisk_, is:

$$SNR_{Whisk}=M_{Whisk}\sqrt{\langle \lambda \rangle T}=2\lambda_{Amplitude}\sqrt{\frac{T}{\lambda }}.$$ (6)

The statistical significance of the modulation was determined by comparing the distributions of all spike phases, N(ϕ_k_|spike) with all whisk phases, N(ϕ_k_), using a Kuiper test [51,90], *i*.*e*., a modification of the Kolmogorov-Smirnov test that accounts for periodic variables.

#### Air-puff

We characterized the modulation in spike rate of each unit in response to air-puff deflections based on a peri-stimulus time histogram of spiking activity aligned to the onset of the stimulus. The histograms were computed by counting the number of spikes in 5 ms bins that were averaged by a 2 ms sliding window. We denote the maximum and the minimum binned spike rates in the first 100 ms after the onset of the stimulus as λ_maximum_ and λ_minimum_, respectively. We computed the background rate, denoted λ_background_ as the mean rate for the 100 ms period that was 150 to 250 ms after the onset of the stimulus. As the response to air-puff typically was observed to be fully contained within the first 100 ms, the statistical significance of the modulation upon air-puff was determined by comparing the distribution of spike times in the first 100 ms post-stimulus to a uniform distribution (Kolmogorov-Smirnov test).

## Supporting Information

S1 Data Data for Fig 2b–2f. (XLSX)

S2 Data Data for Fig 3i and 3j. (XLSX)

S3 Data Data for Fig 3k and 3l. (XLSX)

S4 Data Data for Figs 5d and 6a, 6b
(XLSX)

S5 Data Data for Figs 8e, 9a-9c and 10d, 10e
(XLSX)

S6 Data Data for S4 Fig. (XLSX)

S1 Fig Locations of all identified recording sites in VPM thalamus. The recording sites (blue) were demarked by depositing Chicago Sky Blue dye through the recording pipette at the conclusion of each unit recording (Methods). The red arrowheads point to the detected dye spots. The borders of different thalamic nuclei, including VPM and PO thalamus, as well as ZIv were identified by counterstaining the tissue for cytochrome oxidase activity (brown). These locations and borders are summarized in the 3-dimensional reconstruction shown in Fig 9d and 9e. Each labeled site is associated with an individual “Record ID” that links it to the electrophysiological parameters included as Supplemental Data and plotted in Figs 8e, 9a–9c and 10d, 10e.
(TIF)

S2 Fig Locations of all identified recording sites in PO thalamus. Conventions are as in S1 Fig.
(TIF)

S3 Fig Locations of all identified recording sites in ZIv. Conventions are as in S1 Fig.
(TIF)

S4 Fig Estimate of uncertainty in defining distance to VPM/PO border The uncertainty in the locations of recording sites relative to the VPM/PO border (Fig 9c) in our study is dominated by the inability to precisely identify the border in cytochrome-oxidase sections. This uncertainty varies with the rostro-caudal location of the cell, as the border is less clear rostrally, as well as the quality of the cytochrome-oxidase stain. **(a-f)** To estimate this uncertainty on a cell by cell basis we defined, by visual inspection, the range of locations which would lead to an ambiguous classification between VPM and PO thalamus. Six example cases are shown (black line segments). We define the uncertainty as the width of this range along the line that passes through the recording site and is approximately perpendicular to the VPM/PO border (white line segments). **(g)** Histogram of the uncertainty ranges for all labeled recording sites (median = 80 μm). The raw data for is tabulated in as supplemental information SI Data 6.xlsx.
(TIF)

## Abbreviations

nRt: nucleus reticularis
PrV: principal trigeminal nucleus
PO: posterior medial
SpVC: spinal nucleus caudalis
SpVIc: caudal division of spinal nucleus interpolaris
SpVM: spinal nucleus muralis
SpVIr: rostral division of spinal nucleus interpolaris
VPMdm: dorso-medial division of ventroposterior lateral
ZIv: ventral aspect of zona incerta
