# Supplementary figures and images for: Vibrissa Self-Motion and Touch Are Reliably Encoded along the Same Somatosensory Pathway from Brainstem through Thalamus
Source: PLoS Biol. 2015 Sep 22;13(9):e1002253. doi: 10.1371/journal.pbio.1002253 (PMC4579082; doi:10.1371/journal.pbio.1002253)

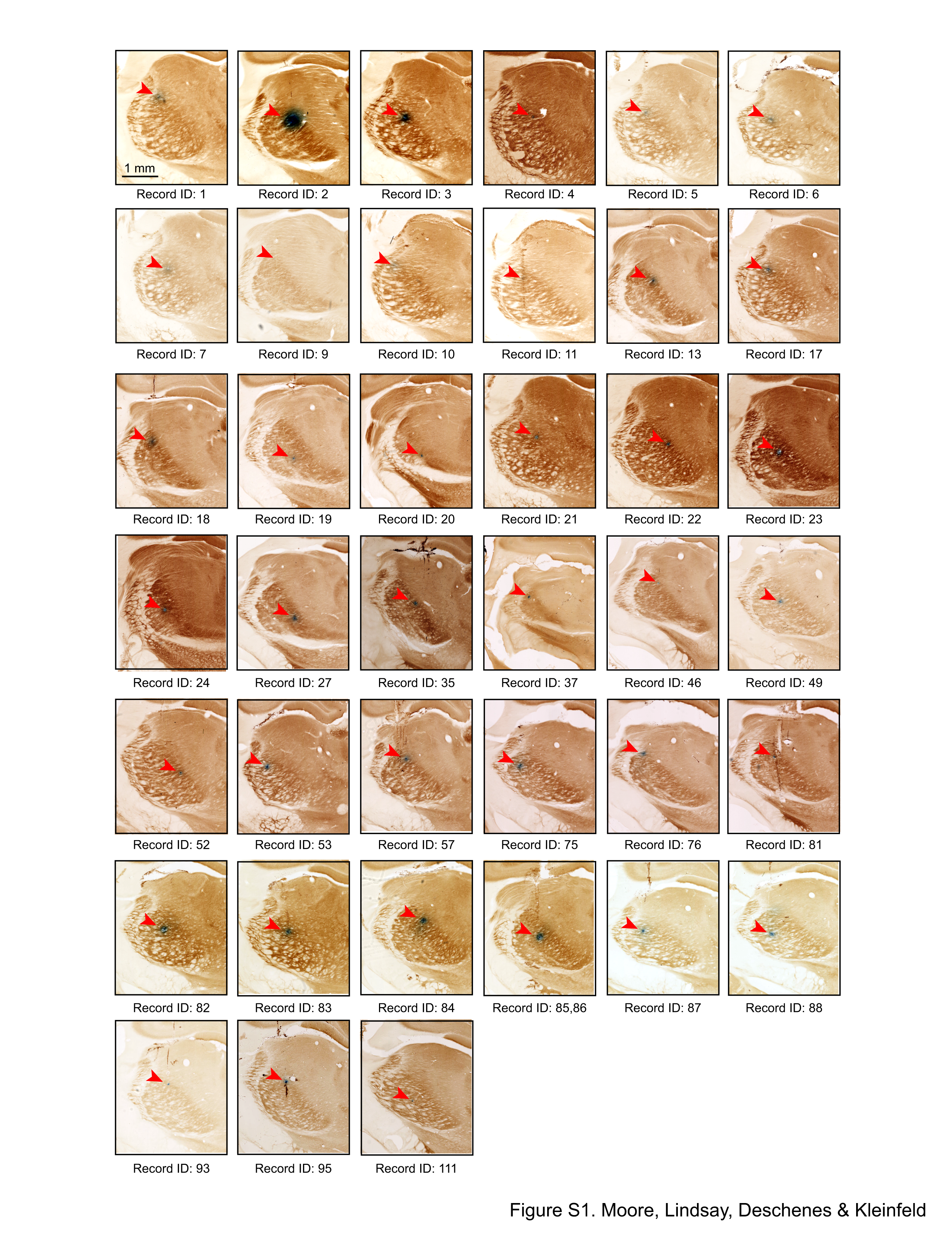

Supplement: S1 Fig — The recording sites (blue) were demarked by depositing Chicago Sky Blue dye through the recording pipette at the conclusion of each unit recording (Methods). The red arrowheads point to the detected dye spots. The borders of different thalamic nuclei, including VPM and PO thalamus, as well as ZIv were identified by counterstaining the tissue for cytochrome oxidase activity (brown). These locations and borders are summarized in the 3-dimensional reconstruction shown in Fig 9d and 9e. Each labeled site is associated with an individual “Record ID” that links it to the electrophysiological parameters included as Supplemental Data and plotted in Figs 8e, 9a–9c and 10d, 10e. (TIF) [file pbio.1002253.s007.tif]

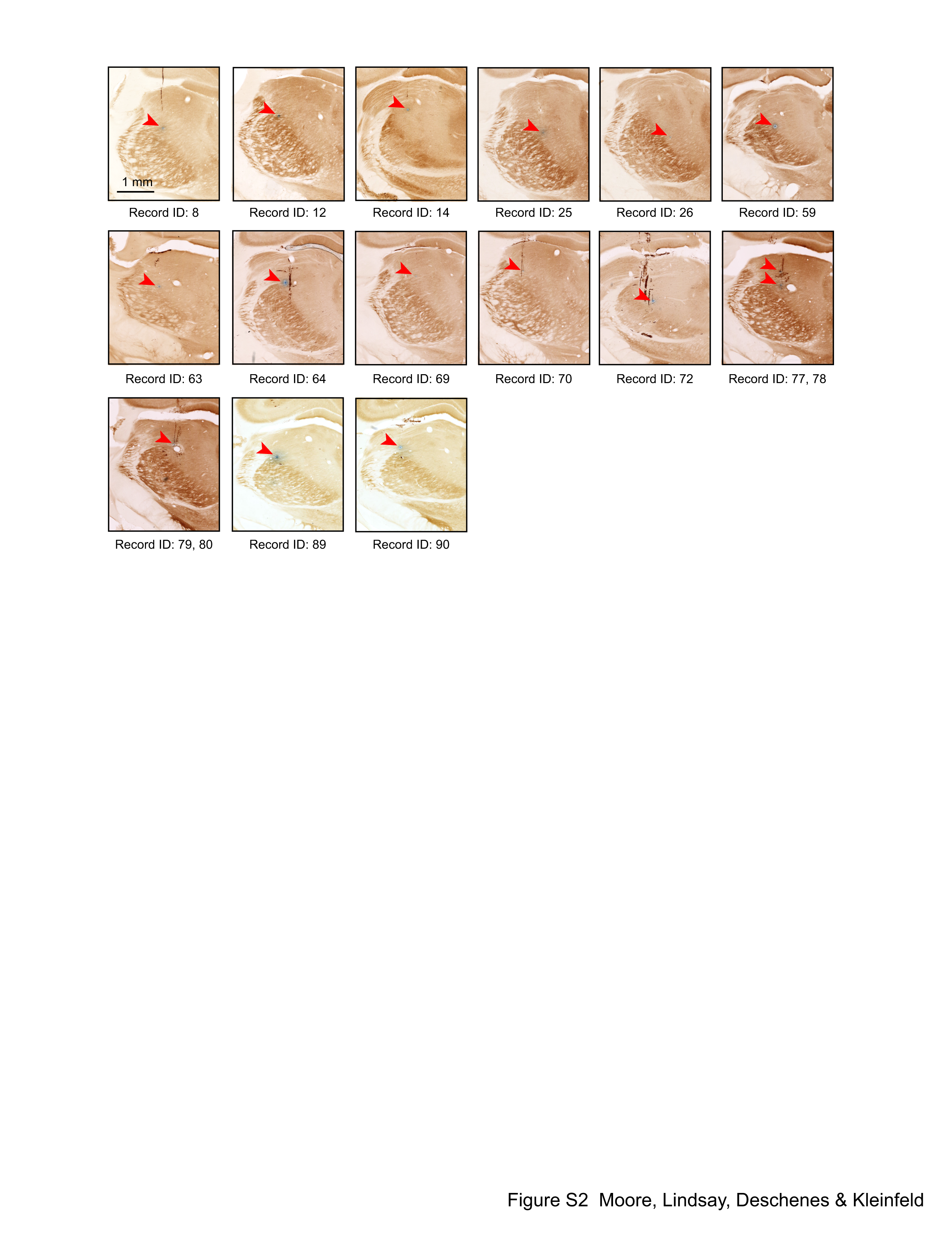

Supplement: S2 Fig — Conventions are as in S1 Fig. (TIF) [file pbio.1002253.s008.tif]

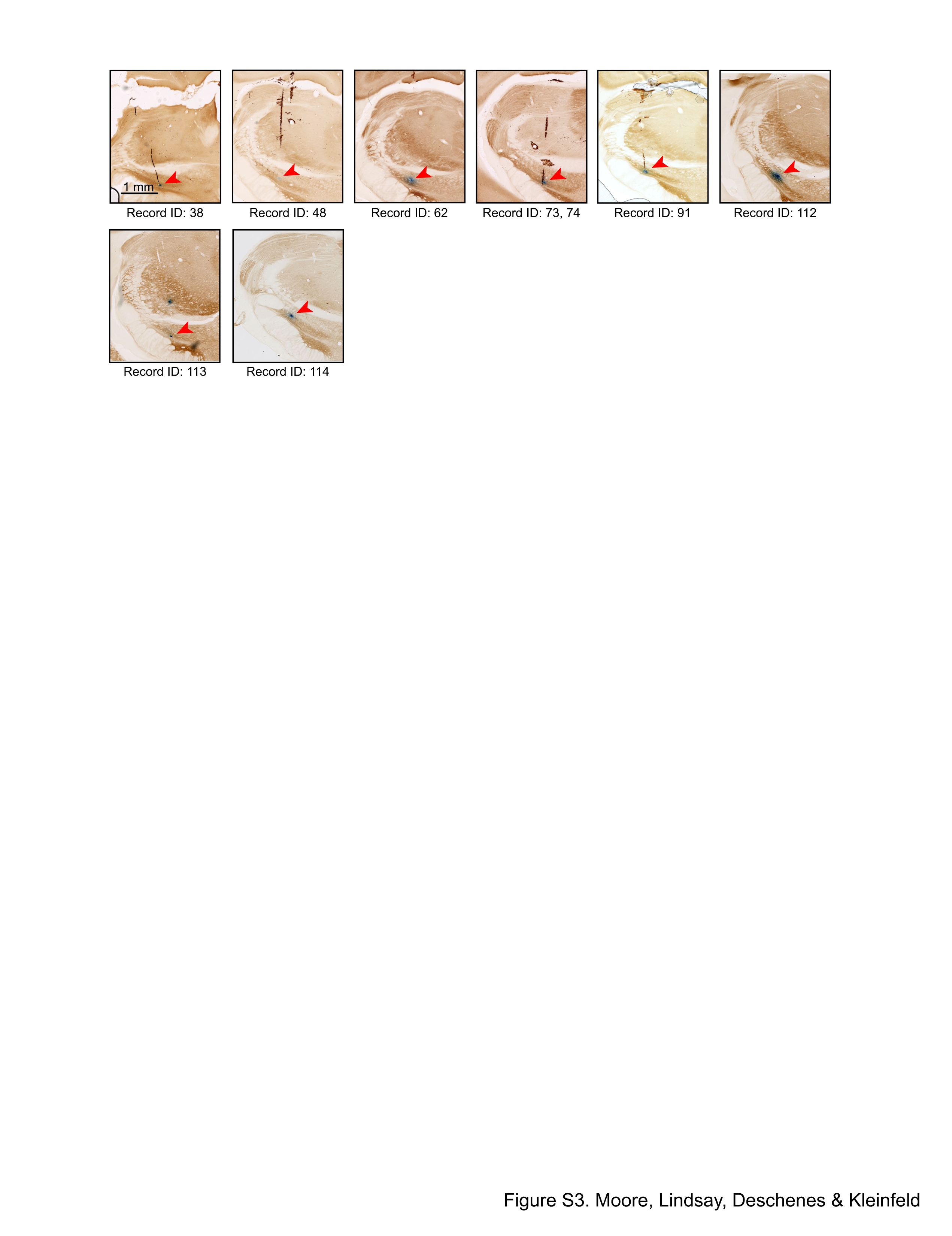

Supplement: S3 Fig — Conventions are as in S1 Fig. (TIF) [file pbio.1002253.s009.tif]

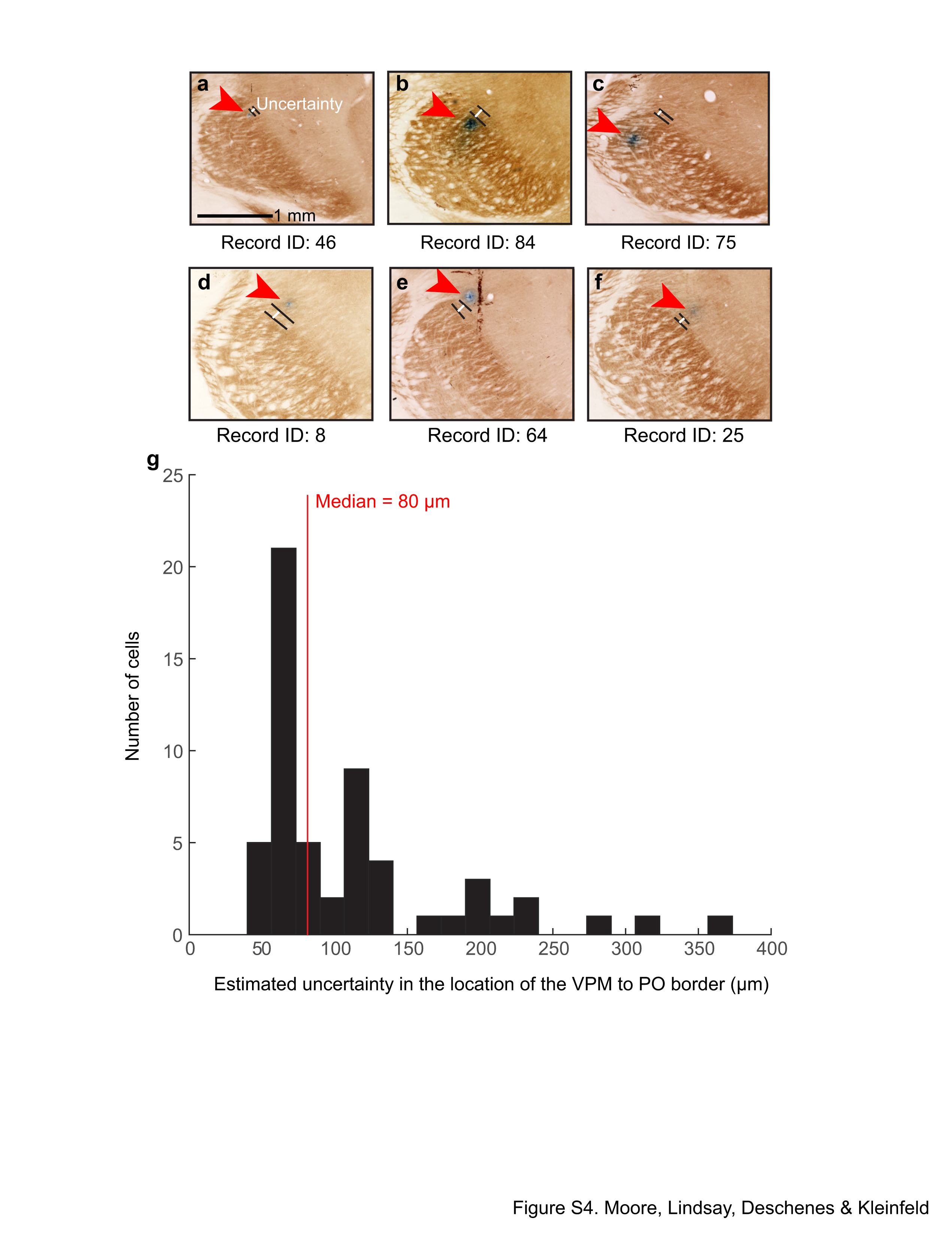

Supplement: S4 Fig — The uncertainty in the locations of recording sites relative to the VPM/PO border (Fig 9c) in our study is dominated by the inability to precisely identify the border in cytochrome-oxidase sections. This uncertainty varies with the rostro-caudal location of the cell, as the border is less clear rostrally, as well as the quality of the cytochrome-oxidase stain. (a-f) To estimate this uncertainty on a cell by cell basis we defined, by visual inspection, the range of locations which would lead to an ambiguous classification between VPM and PO thalamus. Six example cases are shown (black line segments). We define the uncertainty as the width of this range along the line that passes through the recording site and is approximately perpendicular to the VPM/PO border (white line segments). (g) Histogram of the uncertainty ranges for all labeled recording sites (median = 80 μm). The raw data for is tabulated in as supplemental information SI Data 6.xlsx. (TIF) [file pbio.1002253.s010.tif]
